# Remarkable Diversity and Prevalence of Dagger Nematodes of the Genus *Xiphinema* Cobb, 1913 (Nematoda: Longidoridae) in Olives Revealed by Integrative Approaches

**DOI:** 10.1371/journal.pone.0165412

**Published:** 2016-11-09

**Authors:** Antonio Archidona-Yuste, Juan A. Navas-Cortés, Carolina Cantalapiedra-Navarrete, Juan E. Palomares-Rius, Pablo Castillo

**Affiliations:** Instituto de Agricultura Sostenible (IAS), Consejo Superior de Investigaciones Científicas (CSIC), Avenida Menéndez Pidal s/n, 14004 Córdoba, Spain; University of Ostrava, CZECH REPUBLIC

## Abstract

The genus *Xiphinema* includes a remarkable group of invertebrates of the phylum Nematoda comprising ectoparasitic animals of many wild and cultivated plants. Damage is caused by direct feeding on root cells and by vectoring nepoviruses that cause diseases on several crops. Precise identification of *Xiphinema* species is critical for launching appropriate control measures. We make available the first detailed information on the diversity and distribution of *Xiphinema* species infesting wild and cultivated olive in a wide-region in southern Spain that included 211 locations from which 453 sampling sites were analyzed. The present study identified thirty-two *Xiphinema* spp. in the rhizosphere of olive trees, ten species belonging to *Xiphinema americanum*-group, whereas twenty-two were attributed to *Xiphinema* non-*americanum*-group. These results increase our current knowledge on the biodiversity of *Xiphinema* species identified in olives and include the description of four new species (***Xiphinema andalusiense* sp. nov., *Xiphinema celtiense* sp. nov., *Xiphinema iznajarense* sp. nov., and *Xiphinema mengibarense* sp. nov.**), and two new records for cultivate olives (*X*. *cadavalense* and *X*. *conurum*). We also found evidence of remarkable prevalence of *Xiphinema* spp. in olive trees, *viz*. 85.0% (385 out of 453 sampling sites), and they were widely distributed in both wild and cultivated olives, with 26 and 17 *Xiphinema* spp., respectively. Diversity indexes (Richness, Hill´s diversity, Hill´s reciprocal of D and Hill´s evenness) were significantly affected by olive type. We also developed a comparative morphological and morphometrical study together with molecular data from three nuclear ribosomal RNA genes (D2-D3 expansion segments of 28S, ITS1, and partial 18S). Molecular characterization and phylogenetic analyses allowed the delimitation and discrimination of four new species of the genus described herein and three known species. Phylogenetic analyses of *Xiphinema* spp. resulted in a general consensus of these species groups. This study is the most complete phylogenetic analysis for *Xiphinema* non-*americanum-*group species to date.

## Introduction

Soil is most likely one of the more species-rich habitats of terrestrial ecosystems because over one quarter of all living species on Earth are inhabiting the soil [1, 2]. One of the most diverse soil animals are nematodes although they are ubiquitous in all habitats that provide available organic carbon sources [3]. The phylum Nematoda includes species either free-living or parasites of animals or plants. Plant-parasitic nematodes (PPN) comprising about 15% of the total number of nematode species currently known, of which over 4,100 species have been identified as PPN [4, 5]. The fact that new species of PPN are continually being described, combined with PPN gross morphology tends to be highly conserved, likewise the limitations of species concepts, results in an increase of the difficulty in the species identification [6–13]. However, accurate identification of PPN is essential for the selection of appropriate control measures against plant pathogenic species, as well as for a reliable method allowing distinction between species under quarantine or regulatory strategies and a better understanding of their implications in pest control and soil ecology [14]. Integrative taxonomy has been efficiently applied for the accurate diagnostic and identification over the wide range of PPN species [9, 11–13, 15–18].

The most important nematodes economically include endoparasitic species such as the root-knot (*Meloidogyne* spp.) and cyst nematodes (*Heterodera* spp. and *Globodera* spp.), likewise the ectoparasitic nematodes belonging to the family Longidoridae Thorne, 1935 [19]. Dagger nematodes of the genus *Xiphinema* Cobb, 1913 [20] are one of the highest diversified group species of this family [21]. The phytopathological importance of this group of nematodes not only lies in its wide range of host and cosmopolitan distribution but some species of this genus are vectors of several important plant viruses (genus *Nepovirus*, family Comoviridae) that cause significant damage to a wide range of crops [21–26]. Considering the great morphological diversity, the genus *Xiphinema* was divided into two different species groups [14, 22, 27]: i) the *Xiphinema americanum-*group comprising a complex of about 60 species [22, 28]; and ii) the *Xiphinema* non-*americanum-*group which comprises a complex of more than 215 species [14, 17, 18]. Species discrimination in *Xiphinema* is based mainly on classical diagnostic features; however, due to a high degree of intraspecific morphometric variability can lead to overlapping among *Xiphinema* species and increase the risk of species miss-identification [27–29].

Recently, 96 *Xiphinema* species (about 35% of total species) have been characterized molecularly by ribosomal genes (D2-D3 expansion segments of 28S rRNA and ITS1 rRNA and partial 18S), constituting a useful tool for molecular-based species identification [11, 13, 15–18, 28, 30–33]. *Xiphinema* species identification becomes difficult when dealing with morphological closely species that co-occur in a sample or region, as often detected in the Iberian Peninsula [17, 28]. Several authors have highlighted the great diversity of *Xiphinema* spp. detected in the Iberian Peninsula [13, 18, 28, 34–36]. In particular around 40 species of the genus *Xiphinema* have been reported in Spain, mainly associated with woody, ornamental and vegetable plant species [11, 13, 16–18, 37, 38].

Olive, in wild and cultivated forms, is widely distributed in the Mediterranean Basin, and particularly in southern Spain [13, 39–41]. Wild and cultivated olives are hosts and damaged by PPN, including dagger nematodes (*Xiphinema* spp.) [34, 42]. However, little information is available about *Xiphinema* spp. associated with olive trees, except for the recent contributions of Archidona-Yuste *et al*. [13, 18, 28] reporting new species such as *Xiphinema macrodora* Archidona-Yuste *et al*., 2016, *Xiphinema oleae* Archidona-Yuste *et al*., 2016, *Xiphinema plesiopachtaicum* Archidona-Yuste *et al*. 2016, and *Xiphinema vallense* Archidona-Yuste *et al*. 2016 [13, 18, 28]. Therefore, with the aim of deciphering the biodiversity of *Xiphinema* spp. infecting wild and cultivated olives in southern Spain, we surveyed a total of 211 localities at the eight provinces of Andalusia where both olive forms were present. This survey raised 385 populations of *Xiphinema* species, apparently morphologically related to other known *Xiphinema* spp. This prompted us to carry out an integrative taxonomic study to identify the species within this complex genus.

The general objectives of this research was to study the occurrence and abundance of *Xiphinema* species and to test the resemblance between morphological and molecular data within *Xiphinema* species, and the specific objectives were: *i*) to identify the 385 Spanish populations of *Xiphinema* spp. detected in wild and cultivate olives; *ii*) to carry out a molecular characterisation of these *Xiphinema* populations based on sequences of the D2-D3 expansion segments of the 28S nuclear ribosomal RNA gene, the ITS1 of rRNA, and partial 18S rRNA sequences; and *iii*) to study the phylogenetic relationships of *Xiphinema* spp.

## Material and Methods

### Ethics Statement

No specific permits were required for the described fieldwork studies. Permission for sampling the olive orchards was granted by the landowner. The samples from wild olives were obtained in public areas, forests, and other natural areas studied and do not involve any species endangered or protected in Spain. The sites are not protected in any way.

### Soil collection and nematode extraction

**Fig 1:**
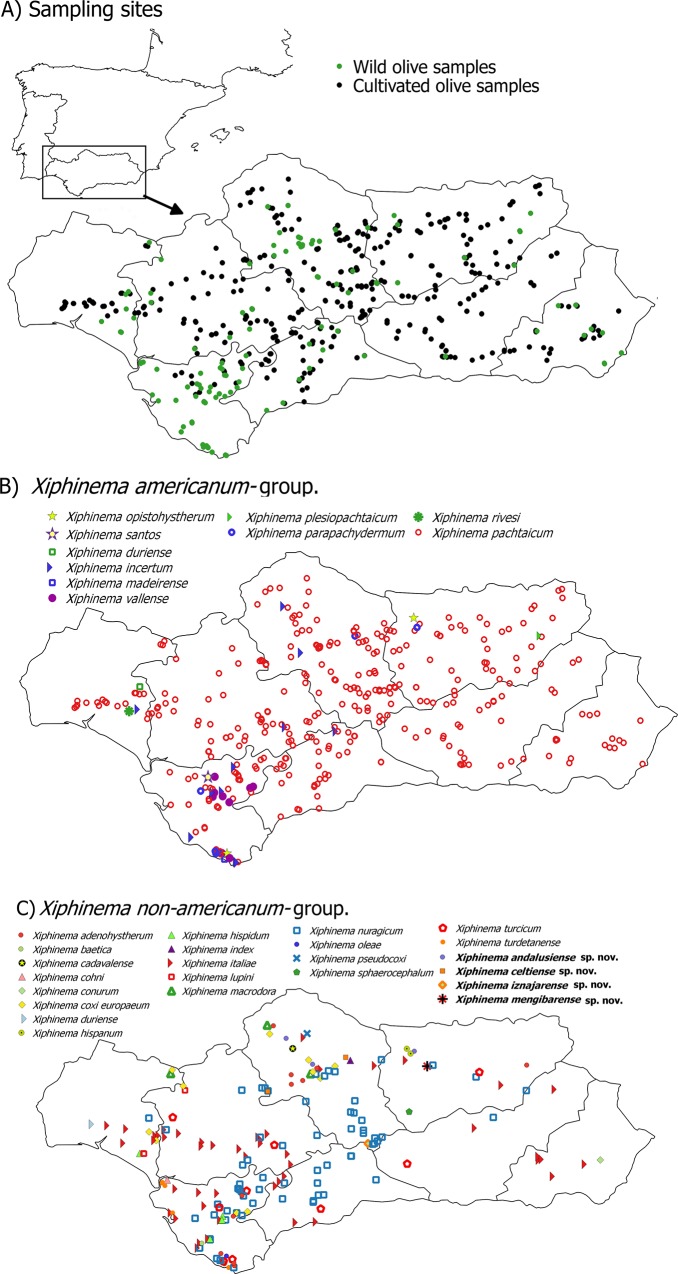
Geographic distribution of dagger nematodes of the genus *Xiphinema* in the present fieldworks on wild and cultivated olive in southern Spain. This map may be similar but not identical to other published maps of Andalusia and is therefore for illustrative purposes only on the sampling sites.

Nematodes were surveyed from 2012 to 2015 during the spring season in wild and cultivate olives growing in Andalusia, southern Spain (Table 1, Fig 1). Soil samples were collected for nematode analysis with a shovel from four to five trees randomly selected in each sampling site. A total of 115 and 338 sampling sites from wild and cultivated olives, respectively, were arbitrarily chosen in the eight provinces of Andalusia where both olive subspecies were present. The number of sampling sites was proportional to the area of wild and cultivated olive in each province (Table 1, Fig 1). Soil samples were collected and analyzed as described by Archidona-Yuste *et al*. [13].

**Table 1:** Taxa sampled for *Xiphinema* species and sequences used in this study.

Species	Sampling site code	Administrative locality	Host-plant	D2-D3	ITS1	Partial 18S	
**1. *X*. *andalusiense* sp. nov.**	AR093	Belmez (Córdoba, Spain)	wild olive	KX244884	KX244921		KX244941
	AR093	Belmez (Córdoba, Spain)	wild olive	-	KX244922		-
	AN419	Andújar (Jaén, Spain)	wild olive	KX244885	KX244923		KX244942
	AN419	Andújar (Jaén, Spain)	wild olive	KX244886	KX244924		-
	AN419	Andújar (Jaén, Spain)	wild olive	KX244887	-		-
	AR108	Villaviciosa (Córdoba, Spain)	wild olive	KX244888	KX244925		-
**2. *X*. *celtiense sp*. *nov*.**	AR083	Peñaflor (Sevilla, Spain)	wild olive	KX244889	KX244926		KX244943
	AR082	Adamuz (Córdoba, Spain)	wild olive	KX244890	KX244927		-
**3. *X*. *iznajarense sp*. *nov*.**	JAO25	Iznájar (Córdoba, Spain)	cultivated olive	KX244891	KX244928		KX244944
	JAO25	Iznájar (Córdoba, Spain)	cultivated olive	KX244892	KX244929		-
**4. *X*. *mengibarense* sp. nov.**	OO3V4	Mengibar (Jaén, Spain)	cultivated olive	KX244893	KX244930		KX244945
	OO3C5	Mengibar (Jaén, Spain)	cultivated olive	KX244894	KX244931		-
	OO3C2	Mengibar (Jaén, Spain)	cultivated olive	KX244895	-		-
**5.** *X*. *adenohystherum* Lamberti *et al*., 1992	AR063	Coto Ríos (Jaén, Spain)	wild olive	KX244896	-		-
	AR078	Almodóvar del Río (Córdoba, Spain)	wild olive	KX244897	-		-
	JAO06	La Granjuela (Córdoba, Spain)	cultivated olive	KX244898	-		-
**6.** *X*. *baetica* Gutiérrez-Gutiérrez *et al*., 2013	AR088	Vejer de la Frontera (Cádiz, Spain)	wild olive	KX244899	-		-
**7.** *X*. *cadavalense* Bravo & Roca, 1995	ST077	Espiel (Córdoba, Spain)	cultivated olive	KX244900	KX244932		KX244946
**8.** *X*. *cohni* Lamberti *et al*., 1992	AR016	Sanlúcar de Barrameda (Cádiz, Spain)	wild olive	KX244901	KX244933		-
**9.** *X*. *conurum* Siddiqi, 1964	ST045	Uleila del Campo (Almería, Spain)	cultivated olive	KX244902	KX244934		KX244947
**10.** *X*. *coxi europaeum* Tarjan 1964	AR092	Alcolea (Córdoba, Spain)	wild olive	KX244903	-		-
	JAO04	Fuente Obejuna (Córdoba, Spain)	cultivated olive	*	-		-
**11.** *X*. *duriense* Lamberti *et al*., 1993	ST002	Gibraleón (Huelva, Spain)	cultivated olive	KX244904	KX244935		-
	AR120	Paterna del Campo (Huelva, Spain)	wild olive	*	-		-
**12.** *X*. *hispanum* Lamberti *et al*., 1992	AR052	Andújar (Jaén, Spain)	wild olive	KX244905	-		-
**13.** *X*. *hispidum* Roca & Bravo, 1994	AR004	Medina Sidonia (Cádiz, Spain)	wild olive	KX244906	-		-
	AR098	Almonte (Huelva, Spain)	wild olive	*	-		-
**14.** *X*. *incertum* Lamberti *et al*., 1983	AR030	Tarifa (Cádiz, Spain)	wild olive	KX244907	-		-
	AR020	Hinojos (Huelva, Spain)	wild olive	KX244908	-		-
	AR104	Mollina (Málaga, Spain)	wild olive	KX244909	-		-
	ST013	Osuna (Seville, Spain)	cultivated olive	*	-		-
**15.** *X*. *index*, Thorne & Allen, 1950	ST123	Adamuz (Córdoba, Spain)	cultivated olive	KX244910	-		-
**16.** *X*. *italiae* Meyl, 1953	AR021	Hinojos (Huelva, Spain)	wild olive	*	-		-
	AR041	Las Tres Villas (Almería, Spain)	wild olive	KX244911	KX244936		-
	AR118	Benahavis (Málaga, Spain)	wild olive	*	-		-
	AR091	Puerto Real (Cádiz, Spain)	wild olive	KX244912	KX244937		-
	ST079	Huévar del Aljarafe (Seville, Spain)	cultivated olive	*	-		-
**17.** *X*. *lupini* Roca & Pereira, 1993	AR099	El Rocío (Huelva, Spain)	wild olive	*	-		-
	AR110	Almadén de la Plata (Sevilla, Spain)	wild olive	*	-		-
**18.** *X*. *macrodora* Archidona-Yuste *et al*., 2016	JAO06	La Granjuela (Córdoba, Spain)	cultivated olive	*	*		*
	JAO47	Santa Olalla del Cala (Huelva, Spain)	cultivated olive	*	*		*
	AR097	Santa Mª de Trassierra (Córdoba, Spain)	wild olive	*	*		*
**19.** *X*. *madeirense* Brown *et al*., 1992	AR031	Tarifa (Cádiz, Spain)	wild olive	*	*		-
**20.** *X*. *nuragicum* Lamberti *et al*., 1992	JAO36	Casarabonela (Málaga, Spain)	cultivated olive	KX244913	-		-
	AR055	San José del Valle (Cádiz, Spain)	wild olive	*	-		-
	JAO79	Úbeda (Jaén, Spain)	cultivated olive	*	-		-
	JAO87	Pedro Martínez (Granada, Spain)	cultivated olive	*	-		-
**21.** *X*. *oleae* Archidona-Yuste *et al*., 2016	AR035	Tarifa (Cádiz, Spain)	wild olive	*	*		*
**22.** *X*. *opisthohysterum* Siddiqi, 1961	AR031	Tarifa (Cádiz, Spain)	wild olive	*	KX244938		-
**23.** *X*. *pachtaicum* (Tulaganov, 1938) Kirjanova, 1951	AR040	Riogordo (Málaga, Spain)	wild olive	*	-		-
	AR073	Castillo de Locubín (Jaén, Spain)	wild olive	*	-		-
	AR042	Tabernas (Almería, Spain)	wild olive	*	-		-
	JAO61	Paterna del Campo (Huelva, Spain)	cultivated olive	*	-		-
**24.** *X*. *parapachydermum* Gutiérrez-Gutiérrez *et al*., 2012	AR035	Tarifa (Cádiz, Spain)	wild olive	KX244914	-		-
	ST122	Adamuz (Córdoba, Spain)	cultivated olive	*	-		-
**25.** *X*. *plesiopachtaicum* Archidona-Yuste *et al*., 2016	AR063	Coto Ríos (Jaén, Spain)	wild olive	*	-		-
**26.** *X*. *pseudocoxi* Sturhan, 1984	AR095	Alcaracejos (Córdoba, Spain)	wild olive	KX244915	KX244939		KX244948
	AR095	Alcaracejos (Córdoba, Spain)	wild olive	KX244916	KX244940		-
**27.** *X*. *santos* Lamberti *et al*., 1993	AR126	Arcos de la Frontera (Cádiz, Spain)	wild olive	*	-		-
**28.** *X*. *sphaerocephalum* Lamberti *et al*., 1992	AR073	Castillo de Locubín (Jaén, Spain)	wild olive	KX244917	-		-
**29.** *X*. *rivesi* Dalmasso, 1969	ST076	Bollullos Par del Condado (Huelva, Spain)	cultivated olive	*	-		-
***30*.** *X*. *turcicum* Luc, 1963	ST090	Santa Cruz del Comercio (Granada, Spain)	cultivated olive	KX244918	-		-
	ST149	Prado del Rey (Cádiz, Spain)	cultivated olive	KX244919	-		-
	ST199	Úbeda (Jaén, Spain)	cultivated olive	*	-		-
	AR124	Sanlúcar la Mayor (Sevilla, Spain)	wild olive	*	-		-
	JAO39	Monda (Málaga, Spain)	cultivated olive	*	-		-
**31.** *X*. *turdetanense* Gutiérrez-Gutiérrez *et al*., 2013	AR090	El Puerto de Sta. María (Cádiz, Spain)	wild olive	KX244920	-		-
	AR017	Sanlúcar de Barrameda (Cádiz, Spain)	wild olive	*	-		-
**32.** *X*. *vallense* Archidona-Yuste *et al*., 2016	AR055	San José del Valle (Cádiz, Spain)	wild olive	*	*		-
	AR027	Tarifa (Cádiz, Spain)	wild olive	*	-		-
	H0003	Hinojos (Huelva, Spain)	cultivated olive	*	-		-

(-) Not obtained or not performed.

(*) Sequenced population but not deposited in GenBank database, since was identical to other sequences of the same species.

Nematodes were extracted from a 500-cm^3^ sub-sample of soil by a modification of Cobb´s decanting and sieving method [43]. Since recovery nematode effectiveness is highest in Cobb´s decanting and sieving method [43, 44], these data were used for prevalence and abundance data analyses. In some samples in which new taxa were detected and more specimens were required for suitable descriptions, additional soil samples were extracted by centrifugal-flotation [45]. The nematode sample processing was carried out as described by Archidona-Yuste *et al*. [13]. PPN from soil samples were identified to genus, and then we focussed on the species delineation of dagger nematodes of the genus *Xiphinema*.

### Diversity indexes

Based on the *Xiphinema* spp. populations detected infesting soils from olives in Andalusia, conventional ecological and diversity indexes were performed in order to evaluate the distribution and changes in the diversity in wild and cultivated olives. In this regard, abundance and prevalence of each *Xiphinema* species identified were estimated. For each sampling site, abundance was calculated as the mean number of *Xiphinema* nematodes per 500 cm^3^ of soil for all samples. The prevalence was computed by dividing the number of samples in which a *Xiphinema* species was detected by the total number of samples and expressed as a percentage.

Several diversity indexes including Hill´s diversity, Hill´s reciprocal of D (Simpson´s dominance index) and Hill´s evenness indexes [46] were calculated according to code indications described by Neher & Darby [47] using the SAS 9.4 software; in addition, Richness index was obtained using principal function implemented in the ‘vegan’ version 2.2–1 package [48] with the R version 3.1.1 software (R Core Development Team). Additionally, abundance and diversity indexes results were subjected to a univariate analysis of variance (ANOVA) and mean values were compared by the Tukey’s test [49] for *P* ≤ 0.05 using the general model procedure of SAS (Statistical Analysis System v. 9.4; SAS Institute, Cary, NC, USA).

### Morphological studies

*Xiphinema* specimens for light microscopy were killed by gentle heat, fixed and examined *Xiphinema* specimens as described by Archidona-Yuste *et al*. and Seinhorst [13, 50]. The morphometric study and drawing of each nematode population was carried out as described in previous papers [13, 14, 22, 27, 51]. All abbreviations used are as defined in Jairajpuri & Ahmad [51]. In addition, a comparative morphological and morphometrical study of type specimens of some species were conducted with specimens kindly provided by Dr. A. Troccoli, from the nematode collection at the Istituto per la Protezione Sostenibile delle Piante (IPSP), Consiglio Nazionale delle Ricerche (CNR), Bari, Italy (*viz*. *Xiphinema cadavalense* Bravo & Roca 1995 [52], and Dr. T. Mateille, from the French Nematode Collection, IRD, Montpellier, France (slides 15368–15376) (*viz*. *Xiphinema conurum* Siddiqi, 1964 [53]. Nematode populations of *Xiphinema* species already described were analysed morphologically and molecularly in this study and proposed as standard and reference populations for each species given until topotype material becomes available and molecularly characterized. Voucher specimens of these described species have been deposited in the nematode collection of Institute for Sustainable Agriculture, IAS-CSIC, Córdoba, Spain.

### DNA extraction, PCR and sequencing

For molecular analyses, in order to avoid mistakes in the case of mixed populations, two live nematodes from each sample were temporary mounted in a drop of 1M NaCl containing glass beads (to avoid nematode crushing/damaging specimens) to ensure specimens conformed to the unidentified populations of *Xiphinema*. Following morphological confirmation, the specimens were removed from the slides and DNA extracted.

Detailed protocols for nematode DNA extraction, PCR and sequencing were applied as described by Castillo *et al*. [54]. The D2-D3 expansion segments of 28S rRNA, ITS1 region, and the portion of the 18S-rRNA were amplified using primers described in previous papers [13, 55–58]. PCR products were purified and sequenced as described by Archidona-Yuste *et al*. [13]. The newly obtained sequences were submitted to the GenBank database under accession numbers indicated on the phylogenetic trees and in Table 1.

### Phylogenetic analysis

D2-D3 expansion segments of 28S rRNA, ITS1, and partial 18S rRNA sequences of different *Xiphinema* spp. from GenBank were used for phylogenetic reconstruction. Outgroup taxa for each dataset were chosen according to previous published data [13, 17, 18]. Multiple alignments of the different genes were made using the Q-INS-i algorithm of MAFFT v. 7.205 [59], strategy FFT-NS-1 with default parameters. Sequence alignments were visualized using BioEdit [60] and edited by Gblocks v0.91b [61] in Castresana Lab server (http://molevol.cmima.csic.es/castresana/Gblocks_server.html) using the less stringent option (Minimum number of sequences for a conserved or a flanking position: 50% of the number of sequences + 1; maximum number of contiguous non-conserved positions: 8; minimum length of a block: 5; allowed gap positions: with half). Percentage similarity between sequences was calculated using a sequence identity matrix in BioEdit. For that, the score for each pair of sequences was compared directly and all gap or place-holding characters were treated as a gap. When position of both sequences has a gap they do not contribute as a difference. Phylogenetic analyses of the sequence data sets were performed based on Bayesian inference (BI) using MrBayes 3.1.2 [62]. The best fitted model of DNA evolution was obtained using jModelTest v. 2.1.7 [63] with the Akaike Information Criterion (AIC). The Akaike-supported model, the base frequency, the proportion of invariable sites, and the gamma distribution shape parameters and substitution rates in the AIC were then used in phylogenetic analyses. BI analyses were performed under GTR+I+G (namely, general time reversible of invariable sites and a gamma-shaped distribution) model for D2-D3 expansion segments of 28S and ITS1 rRNA, and TIM3+I+G (namely, transversional and a transitional of invariable sites and a gamma-shaped distribution) model for the partial 18 S rDNA. These BI analyses were run separately per dataset using four chains for 2 × 10^6^ generations, respectively. The Markov chains were sampled at intervals of 100 generations. Two runs were performed for each analysis. After discarding burn-in samples and evaluating convergence, the remaining samples were retained for further analyses. The topologies were used to generate a 50% majority rule consensus tree. Posterior probabilities (PP) are given on appropriate clades. Trees were visualised using TreeView [64].

### Nomenclatural Acts

The electronic edition of this article conforms to the requirements of the amended International Code of Zoological Nomenclature (ICZN), and hence the new names contained herein are available under that Code from the electronic edition. This published work and the nomenclatural acts it contains have been registered in ZooBank, the online registration system for the ICZN. The ZooBank LSIDs (Life Science Identifiers) can be resolved and the associated information viewed through any standard web browser by appending the LSID to the prefix http://zoobank.org/. The LSID for this publication is: urn:lsid:zoobank.org:pub:CE945C7D-7B14-46DD-8A17-A93A05750590. The electronic edition of this work was published in a journal with an ISSN, and has been archived and is available from the following digital repositories: PubMed Central, LOCKSS.

## Results

### Taxon sampling, abundance, prevalence and diversity indexes of *Xiphinema* spp. in olive

All *Xiphinema* spp. found in this study including specimens of sampling sites used in morphological and/or molecular analyses, are shown in Table 1. In addition, all positive *Xiphinema* spp. and sampling sites are presented in Fig 1. Overall, 32 *Xiphinema* spp. were detected in the rhizosphere of olive trees, ten species belonging to *X*. *americanum*-group, whereas 22 were attributed to *X*. non-*americanum*-group (Table 2). From all *Xiphinema* spp. identified in this study, 26 species were associated with wild olive, whereas seventeen *Xiphinema* species were associated with cultivated olive (Table 1; Fig 1). Eleven *Xiphinema* species occurred in both wild and cultivated olives (*viz*. *X*. *adenohystherum* Lamberti *et al*., 1992 [65], *X*. *coxi europaeum* Tarjan, 1964 [66], *X*. *duriense* Lamberti *et al*., 1993 [67], *X*. *incertum* Lamberti *et al*., 1983 [68], *X*. *italiae* Meyl, 1953 [69], *X*. *macrodora*, *X*. *nuragicum* Lamberti *et al*., 1992 [65], *X*. *pachtaicum* (Tulaganov, 1938) Kirjanova 1951 [70, 71], *X*. *parachydermum* Gutiérrez-Gutiérrez *et al*., 2012 [16], *X*. *turcicum* Luc, 1963 [72] and *X*. *vallense*), while the remaining 21 identified species where present either in wild or cultivated olives only.

**Table 2:** Soil nematode population density (number of specimens) and prevalence (%) of *Xiphinema* spp. in wild and cultivated olives in Andalusia, southern Spain.

Host plant ^a^	Wild olive (W)				Cultivated olive (C)				Global data (W + C)			
Number of samples	115				338				453			
	Density^b^	Min^b^	Max^b^	Prevalence^c^	Density	Min	Max	Prevalence	Density	Min	Max	Prevalence
***Xiphinema* spp.**	**22.8 ± 35.8**	**1**	**350**	**93.9**	**38.1 ± 53.6**	**1**	**414**	**81.7**	**32.6 ± 48.6**	**1**	**414**	**85.0**
***X*. *americanum-*group spp.** ^**d**^	**22.6 ± 23.7**	**1**	**116**	**78.3**	**43.4 ± 57.8**	**1**	**414**	**79.9**	**37.9 ± 51.9**	**1**	**414**	**79.7**
*Xiphinema duriense*	2 ± 0	2	2	0.90	1 ± 0	1	1	0.30	1.3 ± 0.6	1	2	0.44
*Xiphinema incertum*	22.9 ± 11.3	1	42	9.60	38 ± 0	38	38	0.30	24.2 ± 11.6	1	42	4.74
*Xiphinema madeirense*	11 ± 0	11	11	0.90	-	-	-	-	11 ± 0	11	11	0.22
*Xiphinema opisthohysterum*	8.5 ± 7.8	3	14	1.70	-	-	-	-	8.5 ± 7.8	3	14	0.44
*Xiphinema pachtaicum*	22.7 ± 25.0	1	116	58.3	43.9 ± 58.3	1	414	79.4	39.7 ± 54.0	1	414	74.2
*Xiphinema parapachydermum*	28.6 ± 7.8	16	34	4.30	8 ± 0	8	8	0.30	25.2 ± 10.9	8	34	1.32
*Xiphinema plesiopachtaicum*	112 ± 0	112	112	0.90	-	-	-	-	112 ± 0	112	112	0.22
*Xiphinema santos*	9 ± 0	9	9	0.90	-	-	-	-	9 ± 0	9	9	0.22
*Xiphinema rivesi*	-	-	-	-	58 ± 0	58	58	0.30	58 ± 0	58	58	0.22
*Xiphinema vallense*	13.6 ± 12.8	2	37	6.10	14.0 ± 2.9	12	16	0.60	13.7 ± 11.1	2	37	1.99
***X*. *non-americanum-group spp*.** ^**d**^	**23.1 ± 44.5**	**1**	**350**	**70.4**	**21.2 ± 32.2**	**1**	**218**	**25.1**	**22.2 ± 39.2**	**1**	**350**	**36.6**
***Xiphinema andalusiense* sp. nov.**	13.7 ± 8.7	4	21	2.6	-	-	-	-	13.7 ± 8.7	4	21	0.66
***Xiphinema celtiense sp*. nov.**	42.5 ± 55.9	3	82	1.7	-	-	-	-	42.5 ± 55.9	3	82	0.44
***Xiphinema iznajarense sp*. nov.**	-	-	-	-	34 ± 0	34	34	0.30	34 ± 0	34	34	0.22
***Xiphinema mengibarense* sp. nov.**	-	-	-	-	12 ± 0	12	12	0.30	12 ± 0	12	12	0.22
*Xiphinema adenohystherum*	6.2 ± 4.9	1	14	11.3	1 ± 0	1	1	0.30	5.9 ± 4.9	1	14	3.09
*Xiphinema baetica*	1 ± 0	1	1	0.90	-	-	-	-	1 ± 0	1	1	0.22
*Xiphinema cadavalense*	-	-	-	-	1 ± 0	1	1	0.30	1 ± 0	1	1	0.22
*Xiphinema cohni*	32 ± 0	32	32	0.90	-	-	-	-	32 ± 0	32	32	0.22
*Xiphinema conurum*	-	-	-	-	3 ± 0	3	3	0.30	3 ± 0	3	3	0.22
*Xiphinema coxi europaeum*	14.3 ± 28.0	1	88	7.80	1 ± 0	1	1	0.60	11.9 ± 25.6	1	88	2.43
*Xiphinema hispanum*	6.5 ± 7.8	1	12	1.7	-	-	-	-	6.5 ± 7.8	1	12	0.44
*Xiphinema hispidum*	6.6 ± 5.9	1	14	4.30	-	-	-	-	6.6 ± 5.9	1	14	1.10
*Xiphinema index*	-	-	-	-	3 ± 0	3	3	0.30	3 ± 0	3	3	0.22
*Xiphinema italiae*	45.9 ± 97.4	3	350	11.3	20.8 ± 27.1	1	121	9.70	27.6 ± 55.5	1	350	10.2
*Xiphinema lupini*	6.7 ± 4.6	4	12	2.60	-	-	-	-	6.7 ± 4.6	4	12	0.66
*Xiphinema macrodora*	7 ± 0	7	7	0.90	11.0 ± 4.2	8	14	0.60	9.7 ± 3.8	7	14	0.66
*Xiphinema nuragicum*	34.5 ± 37.6	1	134	31.3	26.9 ± 40.5	1	218	11.2	30.7 ± 39.1	1	218	16.3
*Xiphinema oleae*	4 ± 0	4	4	0.90	-	-	-	-	4 ± 0	4	4	0.22
*Xiphinema pseudocoxi*	10 ± 0	10	10	0.90	-	-	-	-	10 ± 0	10	10	0.22
*Xiphinema sphaerocephalum*	15 ± 0	15	15	0.90	-	-	-	-	15 ± 0	15	15	0.22
*Xiphinema turcicum*	2.3 ± 1.3	1	4	1.70	9.4 ± 8.9	1	22	1.50	6.2 ± 7.3	1	22	1.55
*Xiphinema turdetanense*	2.2 ± 1.3	1	4	4.30	-	-	-	-	2.2 ± 1.3	1	4	1.55

^a^ Host plant: W = wild olive; C = cultivated olive.

^b^ Population density was calculated as the mean of *Xiphinema* nematodes per 500 cm^3^ of soil. Average, standard deviation, minimum and maximum levels in fields/host where this genus, group species or species were detected.

^c^ The prevalence was computed by dividing the numbers of samples in which the *Xiphinema* species was observed by the total number of samples and expressed as a percentage

^d^ *Xiphinema* group species established by Tarjan [66]; Loof & Luc, [27]; Lamberti *et al*. [22]; and Coomans *et al*. [14]

(-) not found

**Fig 2:**
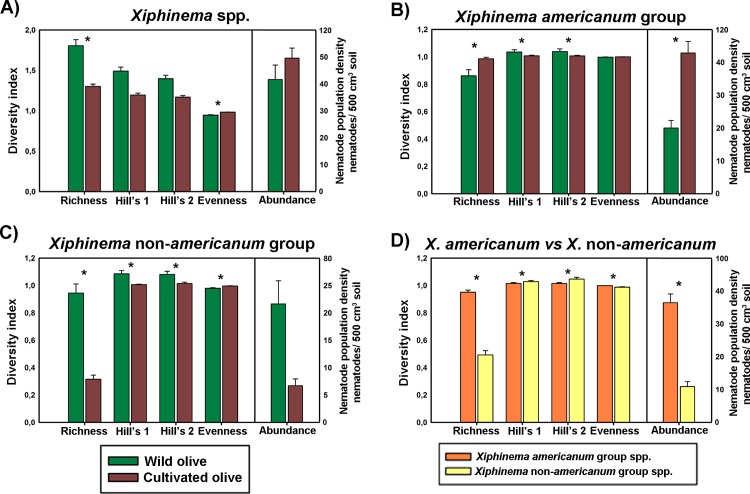
Summary barplot of nematode abundance, Richness, Hill´s diversity (Hill´s 1), Hill´s reciprocal of D (Simpson´s dominance index) (Hill´s 2) and Hill´s evenness diversity indexes derived from results of *Xiphinema* spp. identification in 385 sampling sites of olives orchards (Fig 1) grouped by olive type (wild and cultivated olive) and *X*. *americanum-*group and *X*. non-*americanum-*group species. Error bars indicate the standard error of the mean. Significance = F probability of main effects in ANOVA, according to Tukey´s test [49] for *P <* 0.05.

*Xiphinema* spp. were present in low to high densities (*ca* 33, from 1 to 414 nematodes per 500 cm^3^ of soil) in both wild and cultivated olives, being observed in cultivated olives in higher densities than in wild olives (Table 2 and S1 Table). Nematode abundance of *X*. *americanum*-group species was significantly higher (*P* < 0.01) in cultivated than wild olives (Fig 2B), averaging *ca* 23 *vs* 43 nematodes per 500 cm^3^ of soil for wild and cultivated olives, respectively. On the contrary, nematode density was similar (*P* > 0.05) in both olive types in the *Xiphinema* non-*americanum-*group (Fig 2C), being slightly higher in wild than cultivated olives. In general, *Xiphinema* spp. belonging to *X*. *americanum-*group showed higher densities than species identified within *X*. non-*americanum-*group (*ca* 38 vs 22 nematodes per 500 cm^3^ of soil, respectively) (Table 2 and S1 Table), which resulted in a higher abundance (*P* < 0.001) for *X*. *americanum* group than *X*. non-*americanum-*group species (Fig 2D). On the other hand, the *Xiphinema* species with the highest nematode density was *X*. *pachtaicum* (414 nematodes per 500 cm^3^ of soil), which showed a higher average density in cultivated than wild olives (Table 2 and S1 Table). However, the subsequent species with high nematode density included *X*. *italiae* and *X*. *nuragicum* (350 and 218 nematodes per 500 cm^3^ of soil, respectively), both belonging to *X*. non-*americanum-*group, showing lower average density in cultivated than in wild olives (Table 2 and S1 Table).

The overall prevalence of *Xiphinema* spp. in olive was 85.0% (385 out of 453 sample sites) in Andalusia (Fig 1, Table 2 and S1 Table). However, *Xiphinema* spp. were more prevalent in wild olives (93.9%, 108 out of 115 sampling sites) than cultivated olives (81.7%, 276 out of 338 sampling sites) (Table 2). In addition, the major differences between both olive types occurred in the *Xiphinema* non-*americanum*-group species, being more prevalent in wild than cultivated olives. Nevertheless, prevalence in *X*. *americanum*-group species was similar between both olive types (Table 2). As indicated above for most of the *Xiphinema* spp. identified in this study, the prevalence was higher in wild than cultivated olive except for *X*. *pachtaicum* that was detected in both wild and cultivated olives in all provinces of Andalusia, and being the most prevalent *Xiphinema* species in our study (74.2%, 336 out of 453 sample sites) (Table 2 and S1 Table). The subsequent species with a high prevalence was *X*. *nuragicum* (16.3%, 74 out of 453 sample sites) that was detected in both olive types in the most of the Andalusia provinces, at exception of Almería (Fig 1, Table 1 and S1 Table). Another prevalent *Xiphinema* species belonging also to *X*. non-*americanum-*group was *X*. *italiae* (10.2%, 46 out of 453 sample sites), that was found in both olive types in Almería, Cádiz, Huelva and Málaga provinces, but only in wild olive in Córdoba, Granada, Jaén and Seville provinces (Fig 1, Table 2 and S1 Table).

Several diversity indexes were estimated in our study (Richness, Hill´s diversity, Hill´s reciprocal of D (Simpson´s dominance index), and Hill´s evenness [46]), and tested for differences associated with presence of *Xiphinema* spp. in wild and cultivated olive (Fig 2). Overall, the number of *Xiphinema* spp. detected in each sampling site (Richness index) was significantly affected (*P* < 0.05) by olive type (Fig 2), showing higher values (*P* < 0.001) in wild than cultivated olives (Fig 2A). Similarly, Richness index in *X*. non-*americanum*-group species were significantly higher (*P* < 0.05) in wild than in cultivated olive (Fig 2C), but the opposite occurred in the *X*. *americanum*-group species (Fig 2B). Overall, the Richness index was significantly higher (*P* < 0.001) in *X*. *americanum*-group than in *X*. non-*americanum-*group (Fig 2D). Diversity and dominance diversity indexes (Hill´s 1 and Hill´s 2, respectively) showed similar patterns for both olive types (Fig 2). Thus, significant differences (*P* < 0.05) for both diversity indexes were observed when *Xiphinema* species groups were considered separately (Fig 2B and 2C). On the other hand, the detection of a higher number of species belonging to *X*. non-*americanum-*group linked to the increased presence of prevalent species (*viz*. *X*. *italiae*, *X*. *nuragicum* or *X*. *coxi europaeum*) than *X*. *americanum*-group (Tables 1 and 2) resulted in significant differences (*P* < 0.01) among them when it was considered both olive types (Fig 2D). Evenness diversity showed an inverse trend to that observed in diversity and dominance diversity indexes, with cultivated olives showing higher values (*P* < 0.01) than that of wild olives (Fig 2A and 2C) according to the higher abundance and prevalence (*P* < 0.05) detected in cultivated than wild olives (Table 2 and S1 Table). On the other hand, Evenness index in *X*. *americanum*-group was significantly higher (*P* < 0.001) than that of *X*. non-*americanum*-group species (Fig 2D).

### Taxonomic treatment

Nematoda Linnaeus, 1758 [73]

Dorylaimida Pearse, 1942 [73]

Longidoridae Thorne, 1935 [19]

Longidorinae Thorne, 1935 [19]

*Xiphinema* Cobb, 1913 [20]

***Xiphinema andalusiense* Archidona-Yuste, Navas-Cortés, Cantalapiedra-Navarrete, Palomares-Rius & Castillo, sp. nov.** *urn*:*lsid*:*zoobank*.*org*:*act*:*95E9BE47-B822-4AAF-A11C-50EF7A016137*

Figs 3–5

**Fig 3:**
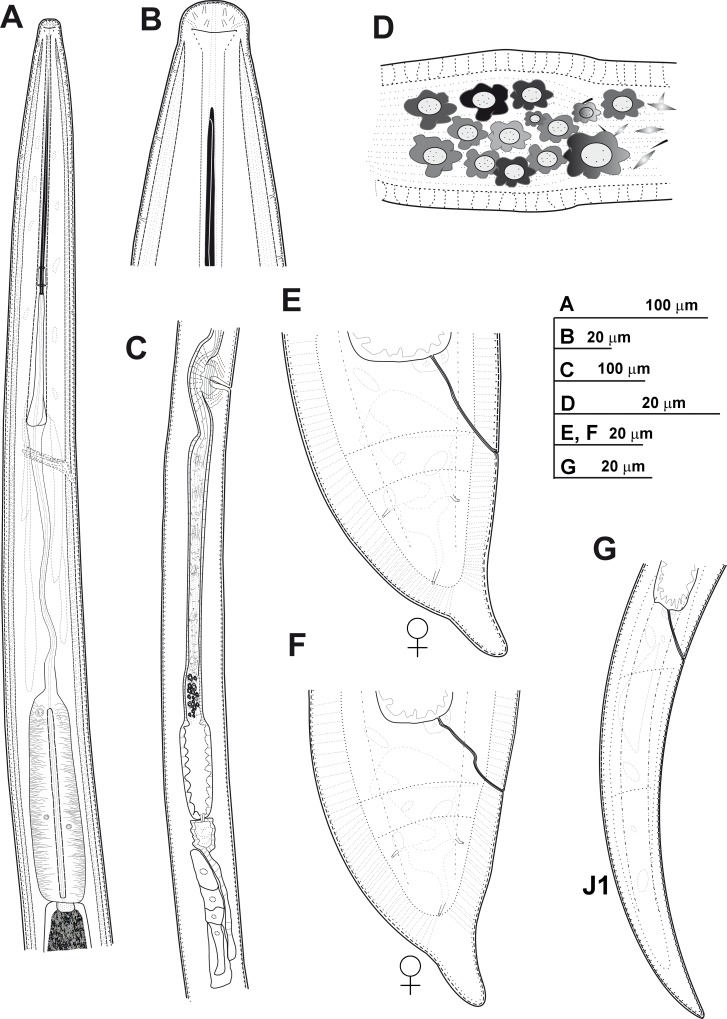
**Line drawings of *Xiphinema andalusiense* sp. nov., female paratypes and first-stage juvenile** A) Pharyngeal region. B) Detail of lip region. C) Posterior female genital branch showing Z-differentiation. D) Detail of Z-differentiation. E-F) Female tails. G) First-stage juvenile tail (J1).

**Fig 4:**
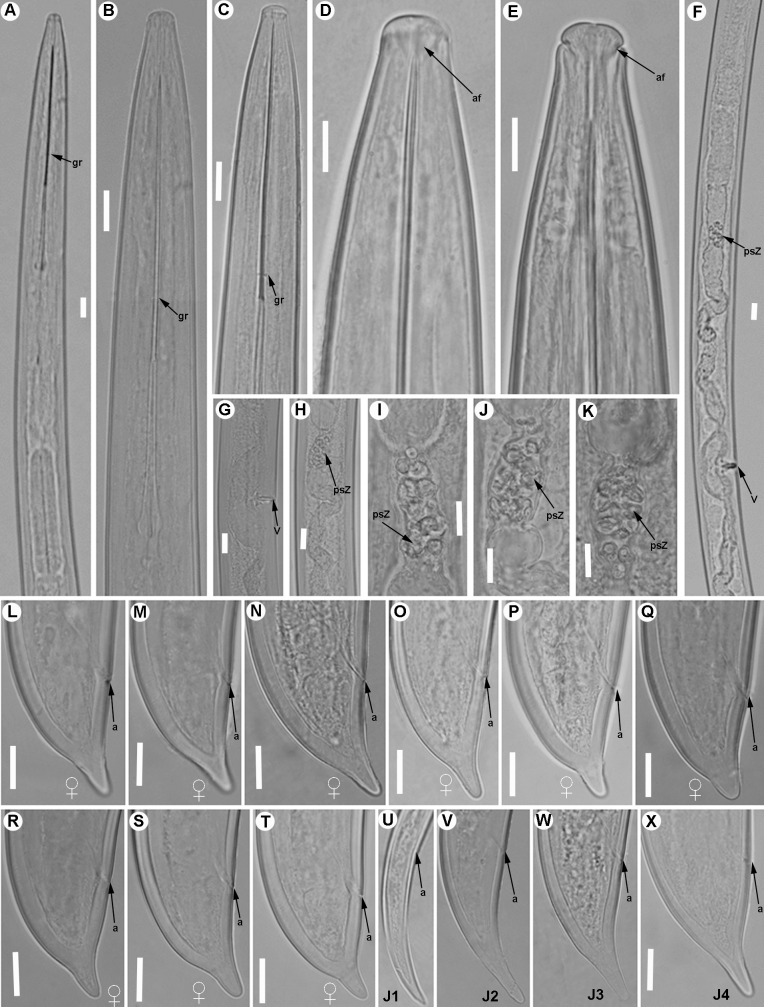
**Light micrographs of *Xiphinema andalusiense* sp. nov., female paratypes and juvenile stages** A) Pharyngeal region. B–E) Female anterior regions. F) Detail of anterior female gonad showing Z-differentiation. G) Vulval region. H) Detail of female genital track showing Z-differentiation. I-K) Z-differentiation. L-T) Female tails. U-X) First-, second-, third-, and fourth-stage juvenile (J1-J4) tails, respectively. Abbreviations: a = anus; cb = crystalloid bodies; gr = guiding-ring; odt = odontostyle; rodt = replacement odontostyle; spi = spiniform structures; spZ = Z-differentiation; v = vulva. Scale bars = 20 μm.

**Fig 5:**
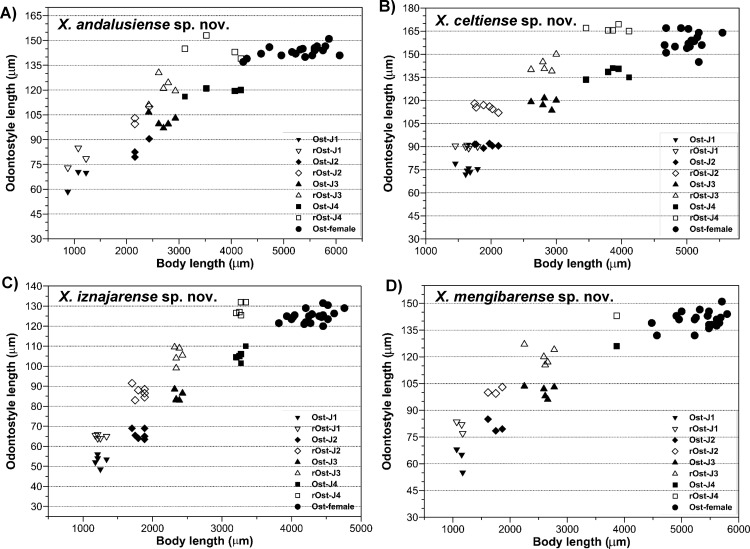
**Relationship between body length and functional and replacement odontostyle (Ost and rOst, respectively) length in all developmental stages from first-stage juveniles (J1) to mature females of:** A) *Xiphinema andalusiense* sp. nov. B) *Xiphinema celtiense* sp. nov. C) *Xiphinema iznajarense* sp. nov. D) *Xiphinema mengibarense* sp. nov.

#### Holotype

Adult female, collected from the rhizosphere of wild olive (*Olea europaea* subsp. *silvestris* (Miller) Lehr) (38°15'10.3"N, 005°09'53.3"W), at Belmez, Córdoba province, Spain; collected by G. Leon Ropero, March 14, 2015; mounted in pure glycerine and deposited in the nematode collection at Institute for Sustainable Agriculture (IAS) of Spanish National Research Council (CSIC), Córdoba, Spain (collection number AR093-2).

#### Paratypes

Female and juvenile paratypes extracted from soil samples collected from the same locality as the holotype; mounted in pure glycerine and deposited in the following nematode collections: Institute for Sustainable Agriculture (IAS) of Spanish National Research Council (CSIC), Córdoba, Spain (collection numbers AR093-5-AR093-7); two females at Istituto per la Protezione Sostenibile delle Piante (IPSP), Consiglio Nazionale delle Ricerche (CNR), Bari, Italy (AR093-8); and one female at USDA Nematode Collection, Beltsville, MD, USA (T-6774p); collected by G. Leon Ropero, March 14, 2015.

#### Diagnosis

*Xiphinema andalusiense* sp. nov. is an apparently parthenogenetic species belonging to morphospecies Group 5 from the *Xiphinema* non-*americanum*-group species [27]. It is characterized by a moderate long body (4.3–6.1 mm), assuming an open C-shaped when heat-relaxed; lip region hemispherical almost continuous or separate from the body contour by a slightly depression, 12.5–15.5 μm wide; a 137.0–151.0 μm long odontostyle; vulva slightly anterior to middle of the body; reproductive system didelphic-amphidelphic with both branches about equally developed having a Z-differentiation in uterus in the form of 11–16 globular bodies in the vicinity of the *pars dilatata uteri*, and small spiniform structures and crystalloid bodies in low number; female tail short, convex-conoid to conical shape with distinctly digitate terminus, and bearing three pairs of caudal pores; c´ ratio (1.0–1.3); and specific D2-D3, ITS1 rRNA and partial 18S rRNA sequences (GenBank accession numbers KX244884-KX244888, KX244921-KX244925, and KX244941-KX244942, respectively). According to the polytomous key of Loof & Luc [27], the new species has the following specific alphanumeric codes (codes in parentheses are exceptions): A4-B2+3-C5a-D5(6)-E5-F5(4)-G3-H2-I3-J4-K6-I1.

#### Etymology

The species epithet refers to the autonomous community from Spain, Andalusia, where the species was detected and moderately distributed.

#### Description of taxa. Female

Body cylindrical and habitus in specimens killed by gentle heat as open C-shape, more curved behind the vulva position, with increasing curvature towards the posterior extremity. Cuticle 3.5–4.0 μm thick at mid-body, but thicker at tail tip, 4.5–8.0 μm wide. Lateral hypodermical chords 18.0–29.0 μm wide at mid body or 29–57% of the corresponding maximum body diameter. Lip region hemispherical, rounded laterally and less so frontally, almost continuous or separated from the body contour by a slightly depression, 12.5–15.5 μm diam. and 5.0–7.5 μm high. Amphidial fovea aperture extending for *ca* 76–88% of lip region diam. and located at *ca* two-thirds of lip region height. Odontostyle long, 1.6–1.9 times longer than odontophore, and the latter with moderate-developed flanges 9.5–12.5 μm wide. Guiding ring with average guiding sheath length of 16.0 μm. Pharynx occupying about 8–15% of body length, consisting of an anterior slender narrow part 346–541 μm long and extending to terminal pharyngeal bulb occupying *ca* 19–27% of total pharyngeal length, 112–139 μm long and 22.5–29.5 μm wide. Glandularium 99.5–119.0 μm long. Nucleus of dorsal pharyngeal gland (DN) located at beginning of basal bulb (10.4–14.3%), ventrosublateral nuclei (SVN) situated *ca* halfway along bulb (46.9–59.4%) (position of gland nuclei calculated as described by Loof & Coomans [74]. In some specimens studied the tip of reserve odontostyle (vestigium) was *ca* 3.5 5 μm in size and directed anteriorly to the isthmus. Cardia conoid, 6.5–14.5 μm long. Prerectum variable in length, 372–783 μm long or 10–19 times anal body diam. Rectum 35.5–47.0 μm long ending in anus as a small rounded slit. Reproductive system didelphic-amphidelphic with branches equally developed and vulva slit-like situated located slightly anterior to mid body. Each branch composed of a 109–212 μm long reflexed ovary and a largely tubular oviduct with enlarged *pars dilatata oviductus* separated from uterus by a well-developed sphincter. Uterus tripartite, comprising a developed *pars dilatata uteri* continuing into a narrower, muscular tube-like portion including a Z-differentiation with weakly muscularised wall and containing 11–16 globular bodies of variable size, each one consisting of a large central portion, irregularly spherical surrounded by a variable number of refractive pieces, and petal shaped (Figs 3 and 4). Low numbers of small spiniform structures and crystalloid bodies along uterus, observed in fresh material in water. Abundant wrinkles observed in uterine wall along uterus, which may be confused as spiniform structures. No sperm was observed in the female genital tract. Ovejector well-developed 41.5–60.0 μm wide, and vagina perpendicular to body axis, 19.5–33.5 μm long or 27–52% of corresponding body diam. in lateral view. In some specimens studied, maturate eggs observed in the tubular part of uterus, 156–183 μm long and 35–43 μm wide. Tail short, varying from convex-conoid to conoid shape with digitate or subdigitate terminus, directed ventrally with respect to the body axis. Distinct terminal blind canal, and in most of specimens studied three caudal pores present on each side.

#### Male

No detected.

#### Juveniles

All four juvenile stages (first-, second-, third- and fourth-stage) were identified using morphological characters such as body length, length of replacement and functional odontostyle (Table 3, Fig 5) [75, 76]. Specifically, J1 were characterised by position of replacement odontostyle just posterior to functional odontostyle, its tip touching or very close to base of functional odontostyle; tail elongate conoid with a slightly dorsal depression at hyaline region and c’ ratio ≥ 3.5 (Figs 3 and 4); and odontostyle length *ca* 66 μm. Tail morphology in second-juvenile stage similar to J1, becoming shorter and stouter than this developmental stage. However, tail morphology in third- and fourth-juvenile stages (except for undeveloped genital structures) similar to that of female, including almost conoid tail shape ending in a digitate terminus (Fig 4), becoming progressively shorter and stouter in each moult, and shorter distance from anterior end to guiding-ring in each moult.

**Table 3:** Morphometrics of females and juvenile developmental stages of *Xiphinema andalusiense* sp. nov. from the rhizosphere of wild olive at Belmez (Córdoba province) southern Spain^a^.

Host/locality, sample code	wild olive, Belmez (Córdoba province) AR093					
Characters/ratios^b^	Holotype	Paratype Females	J1	J2	J3	J4
**n**		19	4	3	6	4
**L (mm)**	5.4	5.3 ± 0.53	1.15 ± 0.23	2.25 ± 0.16	2.72 ± 0.18	3.72 ± 0.49
		(4.2–6.1)	(0.88–1.41)	(2.16–2.43)	(2.42–2.93)	(3.11–4.18)
**a**	84.5	80.3 ± 5.7	52.4 ± 7.1	57.6 ± 3.4	63.3 ± 5.3	73.0 ± 1.6
		(68.7–89.3)	(47.8–62.9)	(54.0–60.8)	(57.9–71.6)	(71.5–75.0)
**b**	9.5	10.0 ± 1.3	4.4 ± 0.7	7.5 ± 0.6	7.3 ± 0.5	7.9 ± 1.8
		(6.8–11.9)	(4.0–5.5)	(6.8–7.9)	(6.3–7.7)	(5.7–9.9)
**c**	127.3	112.9 ± 11.8	16.0 ± 2.1	31.7 ± 4.9	39.8 ± 6.3	66.1 ± 12.6
		(83.7–127.5)	(13.6–18.5)	(27.2–36.8)	(29.5–47.7)	(50.6–81.2)
**c´**	1.0	1.2 ± 0.1	4.7 ± 0.9	2.6 ± 0.4	2.2 ± 0.3	1.5 ± 0.2
		(1.0–1.3)	(3.5–5.5)	(2.2–3.0)	(1.9–2.7)	(1.4–1.8)
**V**	48.0	47.9 ± 1.2	-	-	-	-
		(46.0–50.5)	-	-	-	-
**Odontostyle**	140.0	143.4 ± 3.3	66.0 ± 5.6	84.2 ± 5.7	100.3 ± 3.8	119.1 ± 2.2
		(137.0–151.0)	(58.5–70.5)	(79.5–90.5)	(96.5–106.5)	(116.0–121.0)
**Odontophore**	86.5	82.3 ± 2.9	39.0 ± 4.4	49.3 ± 1.8	66.9 ± 3.5	76.8 ± 1.0
		(76.0–88.5)	(34.5–44.0)	(48.0–50.5)	(64.0–72.0)	(75.5–78.0)
**Total stylet**	226.5	225.7 ± 5.1	-	-	-	-
		(217.5–239.5)	-	-	-	-
**Replacement odontostyle**	-	-	78.8 ± 4.9	104.2 ± 5.3	120.2 ± 7.0	145.0 ± 5.9
	-	-	(73.0–85.0)	(99.5–110.0)	(111.0–130.5)	(139.0–153.0)
**Lip region diam.**	12.5	13.4 ± 1.0	8.1 ± 0.5	9.2 ± 0.6	9.8 ± 1.1	11.5 ± 0.0
		(12.5–15.5)	(7.5–8.5)	(8.5–9.5)	(8.5–11.5)	(11.5–11.5)
**Oral aperture-guiding ring**	138.5	137.3 ± 7.7	48.9 ± 4.4	59.2 ± 7.3	80.8 ± 7.6	108.8 ± 7.4
		(119.5–148.0)	(44.0–53.5)	(51.0–65.0)	(67.5–88.0)	(102.0–118.5)
**Tail length**	42.5	47.0 ± 2.4	71.9 ± 10.1	71.7 ± 7.0	69.5 ± 8.0	57.0 ± 6.4
		(42.5–52.0)	(57.0–79.5)	(66.0–79.5)	(60.0–82.0)	(51.5–63.5)
**J**	14.0	18.6 ± 2.2	10.5 ± 1.3	20.2 ± 6.3	18.8 ± 1.7	21.1 ± 1.8
		(14.0–23.5)	(9.0–11.5)	(14.0–26.5)	(16.5–20.5)	(18.5–22.5)

^a^ Measurements are in μm (except for L) and in the form: mean ± standard deviation (range).

^b^ Abbreviations as defined in Jairajpuri & Ahmad [51]. a, body length/maximum body width; b, body length/pharyngeal length; c, body length/tail length; c', tail length/body width at anus; V (distance from anterior end to vulva/body length) x 100; J (hyaline tail region length).

#### Measurements, morphology and distribution

Morphometric variability is described in Tables 3 and 4 and morphological traits in Figs 3, 4 and 5. In addition to the type locality, *Xiphinema andalusiense* sp. nov. was collected from the rhizosphere of wild olive (*Olea europaea* subsp. *silvestris* (Miller) Lehr) of two localities belonging to Córdoba and Jaén provinces, being one of the new species described in this work which has a broader distribution in Andalusia, concretely on North of Andalusia (Table 1, Fig 1).

**Table 4:** Morphometrics of females of *Xiphinema andalusiense* sp. nov. from the rhizosphere of wild olive at several localities (Córdoba and Jaén provinces) southern Spain^a^.

Host/locality, sample code	wild olive, Villaviciosa (Córdoba province) AR108	wild olive, Andújar (Jaén province) AN419
Characters/ratios^b^	females	females
**n**	1	6
**L (mm)**	4.01	4.72 ± 0.37
		(4.27–5.14)
**a**	64.4	84.9 ± 9.8
		(73.5–97.8)
**b**	10.2	9.6 ± 1.1
		(7.9–10.8)
**c**	83.8	97.1 ± 5.5
		(90.9–105.9)
**c´**	1.2	1.3 ± 0.1
		(1.2–1.3)
**V**	43.5	-
		-
**Odontostyle**	135.0	141.7 ± 4.4
		(137.0–148.0)
**Odontophore**	70.0	79.9 ± 4.7
		(71.0–84.0)
**Total stylet**	205.0	-
		-
**Lip region diam.**	11.5	13.5 ± 0.8
		(12.0–14.5)
**Oral aperture-guiding ring**	124.0	132.5 ± 4.2
		(129.5–141.0)
**Tail length**	48.0	48.6 ± 2.7
		(46.0–53.5)
**J**	15.5	17.3 ± 1.1
		(15.5–18.5)

^a^ Measurements are in μm (except for L) and in the form: mean ± standard deviation (range).

^b^ Abbreviations as defined in Jairajpuri & Ahmad [51]. a, body length/maximum body width; b, body length/pharyngeal length; c, body length/tail length; c', tail length/body width at anus; V (distance from anterior end to vulva/body length) x 100; J (hyaline tail region length).

#### Relationships

According to the polytomous key by Loof & Luc [27] and sorting on matrix codes A (type of female genital apparatus), C (tail shape), D (c´ ratio), E (vulva position), F (body length), and G [total spear length (odontostyle + odontophore)], *X*. *andalusiense* sp. nov. closely resembles *X*. *baetica* Gutiérrez-Gutiérrez *et al*., 2013 [17], *X*. *cadavalense*, and *X*. *turdetanense* Gutiérrez-Gutiérrez *et al*., 2013 [17]. *Xiphinema andalusiense* sp. nov. differs from *X*. *baetica* in few morphological characters including lower a ratio (64.4–89.3 *vs* 91.6–131.2), slightly lower c´ ratio (1.0–1.3 *vs* 1.1–1.8), the presence of spiniform structures or crystalloid bodies along tubular portion of uterus *vs* absent, and the absence *vs* presence of males [17]. On the other hand, *X*. *andalusiense* sp. nov. mainly differs from *X*. *cadavalense* in having a shorter odontostyle and odontophore length (135.0–151.0, 70.0–88.5 *vs* 150.5–164.5 μm, 90.0–111.5 μm, respectively) resulting in a shorter stylet length (215.5–239.5 *vs* 244.5–278.5 μm), a narrower lip region (12.0–15.5 *vs* 14.0–19.5 μm), and higher a and c´ ratios (64.4–89.3, 1.0–1.3 *vs* 454.5–70.9, 0.8–1.2, respectively) [52]. Finally, *X*. *andalusiense* sp. nov. differs from *X*. *turdetanense* in having a slightly longer odontostyle length (137.0–151.0 *vs* 121.0–142.0 μm), a slightly narrower lip region (11.5–15.5 *vs* 14.0–16.0 μm), higher number of globular bodies present in the Z-differentiation (11–16 *vs* 6–8), size and number of spiniform structures presents along tubular part of uterus (low number and smaller *vs* high number and larger), presence of crystalloid bodies along uterus *vs* absence, and the absence *vs* presence of males [17].

In addition, *X*. *andalusiense* sp. nov. is molecularly related to *X*. *macrodora*, but it can be clearly differentiated in having a smaller nematode body and odontostyle length (4.0–6.1 mm, 137.0–151.0 μm *vs* 7.2–8.7 mm, 190.0–206.0 μm, respectively) [18].

#### Molecular divergence of the new species

D2-D3 region of *X*. *andalusiense* sp. nov. (KX244884-KX244888) was 97% similar to *X*. *baetica* (KC567167, KX244899), *X*. *macrodora* (KU171040, KU171042) and *X*. *cadavalense* (KX244900); sequence variation among these species was from 24 to 34 nucleotides and from 3 to 8 indels (Table 5). *Xiphinema andalusiense* sp. nov. showed an intraspecific variation from 0 to 8 nucleotides and no indels. The closest species to *X*. *andalusiense* sp. nov. (KX244921-KX244925) in relation to the ITS1 region were also *X*. *baetica* (KC567156, 89% similar, 119 nucleotides and 28 indels), *X*. *cadavalense* (88% similar, 127 nucleotides and 34 indels), and *X*. *macrodora* (85% similar, 162 nucleotides and 61 indels). Intraspecific variation for this marker was 44 nucleotides and 23 gaps amongst the five studied populations (Table 5). Finally, the partial 18S region of *X*. *andalusiense* sp. nov. showed high similarity values (99%), with several *Xiphinema* spp. such as *X*. *baetica* (KC567148-KC567149), X. *cadavalense* (KX244932), *X*. *macrodora* (KU171050) and *X*. *coxi europaeum* (KC567153).

**Table 5:** Identity matrix, percentage (%) of identical residues between (indels included) rDNA sequences amongst Xiphinema species. Above diagonal D2-D3 expansion segments of 28S rRNA and below diagonal internal transcribed spacer 1 (ITS1) region*.

		*Xiphinema* spp.																	
*Xiphinema* spp.	1	2	3	4	5	6	7	8	9	10	11	12	13	14	15	16	17	18	19
1. *X*. *andalusiense* sp. nov.*		49	49	48	48	87	87	49	42	81	-	80	48	47	48	82	81	47	-
**2. *X*. *celtiense* sp. nov.**	86		85	80	79	49	50	87	58	50	-	47	81	78	72	50	48	70	-
**3. *X*. *iznajarense* sp. nov.**	87	94		79	79	48	49	86	57	49	-	48	81	77	72	49	48	70	-
**4 *X*. *mengibarense* sp. nov.**	86	93	93		76	49	49	82	59	50	-	48	76	75	73	49	48	70	**-**
5. *X*. *adenohystherum*	88	**95**	**96**	94		48	49	80	56	49	-	47	84	81	73	48	48	72	-
6. *X*. *baetica*	**98**	86	87	87	88		88	49	42	82	-	80	48	47	48	84	80	45	**-**
7. *X*. *cadavalense*	97	86	86	86	87	98		50	42	84	-	83	48	47	49	83	84	46	-
8. *X*. *cohni*	87	**96**	84	93	85	87	86		58	50	-	48	81	78	72	49	48	70	-
9. *X*. *conurum*	84	88	88	88	88	84	84	88		43	-	42	57	56	54	41	42	61	-
10. *X*. *coxi europaeum*	**96**	86	86	86	87	86	86	86	84		-	83	48	48	48	78	82	46	-
11. *X*. *gersoni*	87	**95**	**95**	94	**97**	87	86	**95**	88	86		-	-	-	-	-	-	-	-
12. *X*. *globosum*	**96**	86	88	87	88	86	86	86	85	**96**	87		46	46	47	77	84	46	**-**
13. *X*. *hispanum*	87	95	**96**	93	98	87	86	**95**	88	87	**96**	88		83	76	48	47	72	-
14. *X*. *hispidum*	88	97	**95**	94	95	88	87	**97**	89	87	**96**	88	**95**		74	48	46	73	**-**
15. *X*. *italiae*	87	93	93	94	94	87	87	93	88	87	**95**	87	**95**	94		47	47	70	-
16. *X*. *macrodora*	**97**	87	87	87	88	**97**	**97**	87	84	**96**	87	**96**	87	88	88		77	45	-
17. *X*. *pseudocoxi*	94	86	87	87	88	**95**	**95**	86	85	**96**	87	**98**	87	88	87	**96**		**45**	**-**
18. *X*. *pyrenaicum*	87	93	93	94	94	87	86	93	89	86	94	87	94	94	94	87	87		**-**
19. *X*. *sphaerocephalum*	87	94	85	94	94	87	87	94	89	87	94	87	**95**	**95**	94	88	87	94	-

* Similarity between sequences ≥ 95% are in bold letters.

(-) Sequences not available or comparison not carried out because of low homology between sequences.

***Xiphinema celtiense* Archidona-Yuste, Navas-Cortés, Cantalapiedra-Navarrete, Palomares-Rius & Castillo, sp. nov.** *urn*:*lsid*:*zoobank*.*org*:*act*:*17E565E4-18E8-4D60-AA57-55253F3E257E*

Figs 5–7

**Fig 6:**
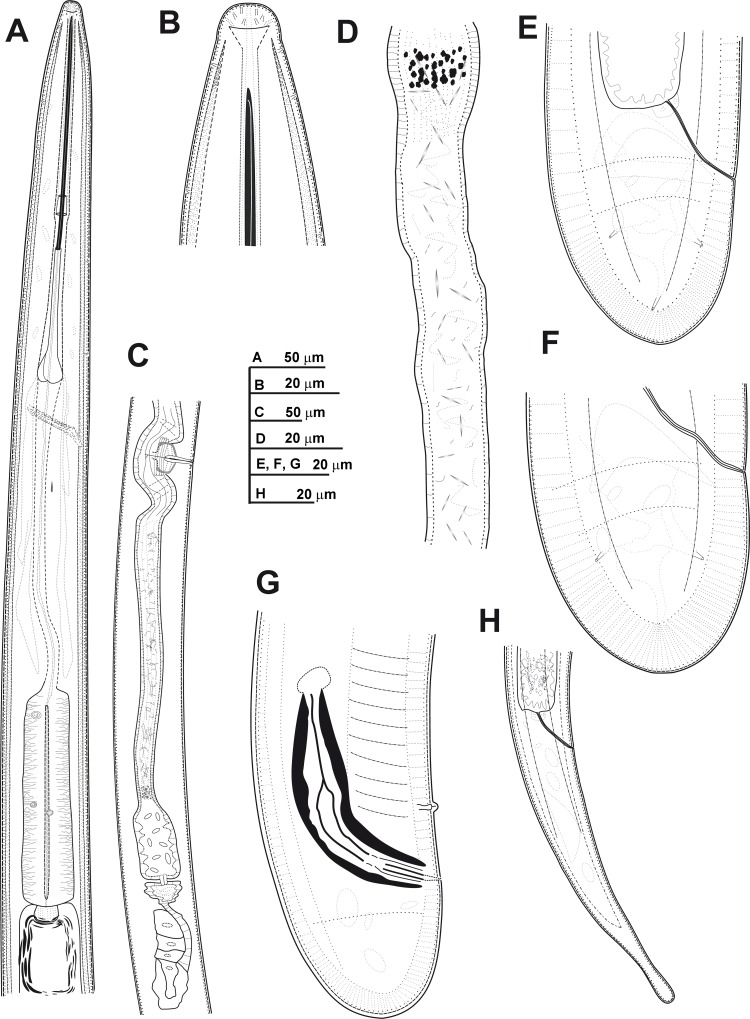
**Line drawings of *Xiphinema celtiense* sp. nov., female paratypes, male and first-stage juvenile** A) Pharyngeal region. B) Detail of lip region. C) Posterior female genital branch showing Z-differentiation. D) Detail of Z-differentiation. E-F) Female tails. G) Male tail. H) First-stage juvenile tail (J1).

**Fig 7:**
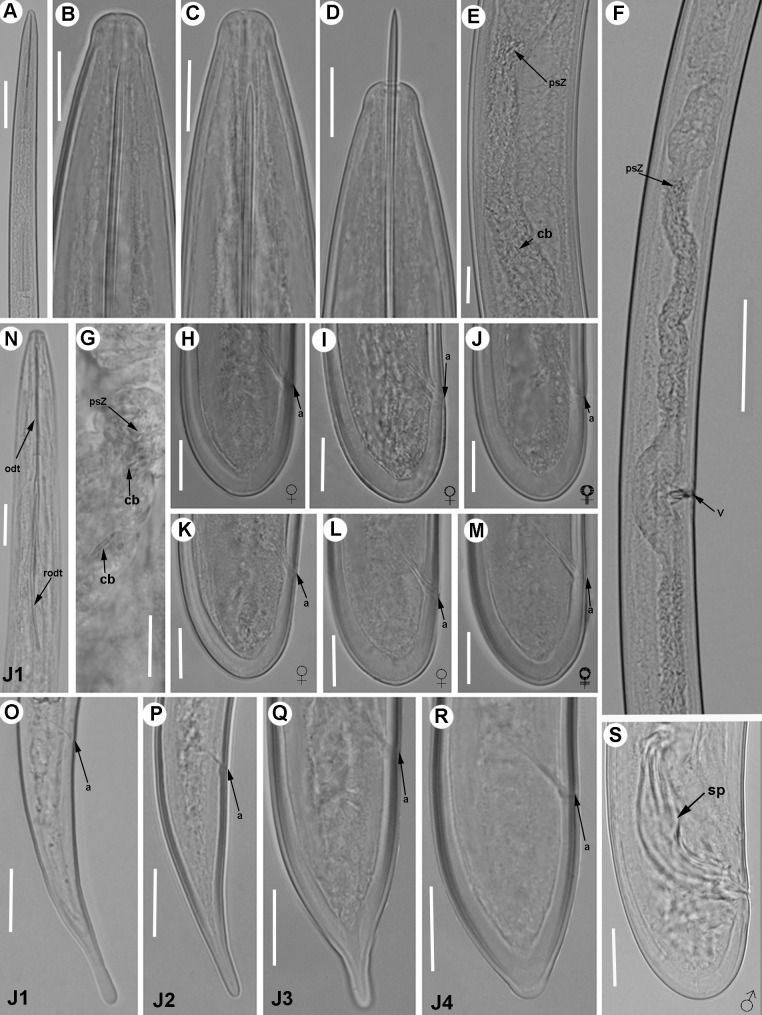
**Light micrographs of *Xiphinema celtiense* sp. nov., female paratypes, male and juvenile stages** A) Pharyngeal region. B–D) Female anterior regions. E) Detail of female genital track showing Z-differentiation. F) Detail of anterior female gonad showing Z-differentiation. G) Z-differentiation. H-M) Female tails. N) Detail of first-stage anterior region. O-R) First-, second-, third-, and fourth-stage juvenile (J1-J4) tails, respectively. S) Male tail with detail of spicules. Abbreviations: a = anus; cb = crystalloid bodies; gr = guiding-ring; odt = odontostyle; rodt = replacement odontostyle; sp = spicules; spZ = Z-differentiation; v = vulva. Scale bars = 20 μm.

#### Holotype

Adult female, collected from the rhizosphere of wild olive (*Olea europaea* subsp. *silvestris* (Miller) Lehr) (38°02'50.9"N, 004°32'52.8"W), at Peñaflor, Seville province, Spain; collected by A. Archidona-Yuste, April 22, 2014; mounted in pure glycerine and deposited in the nematode collection at Institute for Sustainable Agriculture (IAS) of Spanish National Research Council (CSIC), Córdoba, Spain (collection number AR083-01).

#### Paratypes

Female, male and juvenile paratypes extracted from soil samples collected from the same locality as the holotype; mounted in pure glycerine and deposited in the following nematode collections: Institute for Sustainable Agriculture (IAS) of Spanish National Research Council (CSIC), Córdoba, Spain (collection numbers AR083-03-AR083-06); two females and one juvenile at Istituto per la Protezione Sostenibile delle Piante (IPSP), Consiglio Nazionale delle Ricerche (CNR), Bari, Italy (AR083-22); two females and two juveniles at Royal Belgian Institute of Natural Sciences, Brussels, Belgium (RIT 852); and two females and two juveniles at USDA Nematode Collection, Beltsville, MD, USA (T-6775p); collected by A. Archidona-Yuste, April 22, 2014.

#### Diagnosis

*Xiphinema celtiense* sp. nov. is a *Xiphinema* non-*americanum*-group species belonging to morphospecies Group 5 *sensu* Loof & Luc [27]. It is an apparently parthenogenetic species characterized by a moderate long body (4.7–5.5 mm), assuming an open C-shaped when heat-relaxed; lip region hemispherical, both laterally and frontally rounded and separated from body contour by a slight depression, 13.5–16.0 μm wide; long odontostyle (145.0–167.0 μm); vulva situated at mid body; reproductive system didelphic-amphidelphic having both branches about equally developed, Z-differentiation containing almost 15 granular bodies, uterus tripartite with small crystalloid bodies in low number, and presence of prominent wrinkles in the uterine wall that may be confused with spiniform structures; female tail short, varying from hemispherical to convex-conoid shape, bearing two or three pairs of caudal pores; c´ ratio (0.8–1.0); males extremely rare, only one male was found, with moderately long spicules (74.0 μm) and 5 ventromedian supplements; and specific D2-D3, ITS1 rRNA and partial 18S rRNA sequences (GenBank accession numbers KX244889-KX244890, KX244926-KX244927, and KX244943, respectively). According to the polytomous key of Loof & Luc [27], the new species has the following specific alphanumeric codes (codes in parentheses are exceptions): A4-B2-C7-D6-E6-F5-G34-H2-I3-J7-K2-I1.

#### Etymology

The species name is derived from originating Roman city of Peñaflor, ‘*Celti’*, where the type specimens were collected.

#### Description of taxa. Female

Body cylindrical, with open C-shaped upon fixation. Cuticle 2.5–4.0 μm wide at mid-body, but thicker at tail tip, 6.5–11.0 μm wide. Lateral hypodermical chords visible throughout the length of the body, occupying about 23% of the corresponding maximum body diameter. Lip region hemispherical, both frontally and laterally rounded, slightly offset from body contour by a depression, 14.3 ± 0.8 (13.5–16.0) μm wide and 7.2 ± 1.4 (4.5–9.5) μm high. Amphidial fovea aperture extending for *ca* 58–78% of lip region diam. Guiding ring with average guiding sheath length of 15.5 μm. Odontostyle long, 1.4–1.8 times longer than odontophore, and the latter with well-developed flanges 13.0–16.5 μm wide. Pharynx very long occupying about 10–14% of body length, consisting of an anterior slender narrow part 379–510 μm long and extending to pharyngeal bulb, 126.0–168.0 μm long and 22.5–36.0 μm wide. Glandularium 110–155 μm long. Nucleus of dorsal pharyngeal gland (DN) located at beginning of basal bulb (11.5–16.1%), ventrosublateral nuclei (SVN) situated *ca* halfway along bulb (50.5–62.3%) (position of gland nuclei calculated as described by Loof & Coomans [74]. Vestigium small (tip of reserve odontostyle), 3 μm long, observed in all specimens studied in anterior region of slender part of pharynx. Cardia conoid, 8.5–17.5 μm long. Prerectum variable in length, 517–805 μm long, reaching about 10–16% of nematode body from the anus to anterior part. Rectum 36.5–44.0 μm long ending in anus as a small rounded slit. Reproductive system didelphic-amphidelphic with branches about equally developed. Each branch composed of an ovary 113–184 *μ*m long, a reflexed oviduct with well-developed *pars dilatata oviductus* separated from uterus by a well-developed sphincter. Uterus tripartite composed of *pars dilatata uteri* followed by a tubular part containing in the proximal part a well-developed Z-differentiation with weakly muscularised wall, comprising 12–19 small granular bodies similar in size (Figs 6, 7F and 7G). Small crystalloid bodies similar in size and lower in number, mixed with abundant wrinkles from uterine wall, which may be confused as spiniform structures, distributed over the entire length of the tube-like portion of uterus (Figs 6 and 7). In some specimens studied and in a proximal part of *pars dilatata uteri* spindle shaped sperm cells were observed. Ovejector well-developed 46.0–61.5 μm wide, and vagina perpendicular to body axis, 20.0–29.5 μm long or 27–42% of corresponding body diam. in lateral view. Vulva slit-like, situated slightly posterior the mid-body region. Tail short, always shorter than anal body diam., varying in shape from hemispherical to convex-conoid with rounded terminus, and bearing two or three caudal pores present on each side.

#### Male

Extremely rare, only one male specimen was found in type locality. Male genital tract diorchic with testes containing multiple rows of different stages of spermatogonia. Tail short, convex-conoid with a broadly rounded terminus and thickened outer cuticular layer. Spicules moderately long and slightly curved ventrally; lateral guiding pieces more or less straight or with curved proximal end. One pair of adanal and 4 mid-ventral supplements.

#### Juveniles

All four juvenile stages (first-, second-, third- and fourth-stage) were identified using morphological characters such as body length, length of replacement and functional odontostyle (Table 6, Fig 5) [75, 76]. Specifically, J1 were characterised by position of replacement odontostyle just posterior to functional odontostyle, its tip touching or very close to base of functional odontostyle; tail conical elongate, ending in a knob-like expansion, more or less developed, separated from the anterior part of the tail by a depression more or less marked, but giving to the tail a very characteristic profile (Figs 6 and 7); c’ ratio ≥ 4.0; and odontostyle length *ca* 75 μm. Tail morphology of second-stage juvenile similar to J1 expect to absence of knob-like expansion, and tail conoid and subdigitate with rounded terminus for third-stage juvenile. In J4 tail conoid with a short bulge rounded terminus (Fig 7). All juvenile developmental stages with tail becoming progressively shorter and stouter in each moult, and shorter distance from anterior end to guiding-ring in each moult (Table 6, Fig 7).

**Table 6:** Morphometrics of females, males and juvenile stages of *Xiphinema celtiense* sp. nov. from the rhizosphere of wild olive at several localities (Córdoba and Sevilla provinces) southern Spain^a^.

Host/locality, sample code		Peñaflor (Sevilla, Spain) AR083						Adamuz (Córdoba, Spain) AR082
Characters/ratios^b^	Holotype	Paratype Females	Paratype Males	J1	J2	J3	J4	Female
**n**		20	1	6	6	6	6	3
**L (mm)**	5.0	5.0 ± 0.22	4.8	1.64 ± 0.11	1.92 ± 0.14	2.81 ± 0.14	3.76 ± 0.29	5.08 ± 0.32
		(4.7–5.5)		(1.46–1.80)	(1.75–2.11)	(2.61–3.00)	(3.36–4.11)	(4.7–5.4)
**a**	69.3	72.5 ± 3.9	78.3	50.9 ± 2.7	61.5 ± 4.4	63.3 ± 7.6	67.3 ± 5.9	69.2 ± 7.5
		(67.4–81.0)		(48.8–56.1)	(54.7–67.0)	(56.8–75.0)	(59.8–74.3)	(64.8–77.8)
**b**	8.1	8.1 ± 0.5	7.8	5.7 ± 1.2	4.8 ± 0.7	5.8 ± 0.3	6.5 ± 0.8	8.2 ± 0.3
		(7.0–9.4)		(4.2–7.3)	(3.8–5.8)	(5.4–6.2)	(5.8–7.8)	(8.0–8.5)
**c**	109.2	111.2 ± 11.8	132.0	18.4 ± 1.6	25.2 ± 2.9	39.2 ± 4.4	76.1 ± 5.2	109.4 ± 10.6
		(100.7–143.9)		(15.6–20.3)	(22.2–28.8)	(33.1–44.4)	(68.4–82.4)	(97.5–117.9)
**c´**	0.9	0.9 ± 0.1	0.8	4.2 ± 0.3	3.3 ± 0.4	2.1 ± 0.2	1.1 ± 0.1	0.9 ± 0.1
		(0.8–1.0)		(4.0–4.3)	(2.7–3.7)	(1.8–2.5)	(0.9–1.3)	(0.8–0.9)
**V or T**	50.5	51.1 ± 1.1	61.8	-	-	-	-	53.2 ± 1.6
		(50.0–53.5)		-	-	-	-	(52.0–55.0)
**Odontostyle**	148.0	158.4 ± 6.1	162.0	75.1 ± 2.4	90.8 ± 1.1	116.5 ± 5.0	137.9 ± 3.0	167.3 ± 2.9
		(145.0–167.0)		(72.0–76.0)	(89.0–92.0)	(108.0–121.5)	(133.5–141.0)	(164.0–169.0)
**Odontophore**	93.0	93.4 ± 3.2	99.5	51.3 ± 5.3	65.0 ± 1.7	76.0 ± 3.2	85.1 ± 3.6	92.5 ± 2.0
		(89.0–103.0)		(43.5–58.0)	(63.0–67.0)	(72.0–81.0)	(81.0–90.5)	(90.5–94.5)
**Total stylet**	241.0	251.8 ± 5.9	261.5	-	-	-	-	-
		(241.0–260.5)		-	-	-	-	
**Replacement odontostyle**	-	-	-	90.2 ± 2.0	115.5 ± 2.1	141.8 ± 5.0	166.7 ± 1.7	259.8 ± 4.7
				(89.0–91.0)	(112.0–118.0)	(136.0–150.0)	(165.0–169.5)	(254.5–263.5)
**Lip region diam.**	14.0	14.3 ± 0.8	14.5	9.4 ± 0.2	10.2 ± 0.4	11.5 ± 0.3	12.4 ± 0.7	15.3 ± 0.8
		(13.5–16.0)		(9.0–9.5)	(9.5–10.5)	(11.0–12.0)	(11.5–13.5)	(14.5–16.0)
**Oral aperture-guiding ring**	138.0	143.8 ± 6.1	142.0	57.5 ± 5.8	81.8 ± 5.3	101.8 ± 5.1	119.1 ± 10.7	149.0 ± 7.2
		(132.0–155.0)		(51.0–59.0)	(77.0–92.0)	(95.5–107.0)	(107.0–134.0)	(141.0–155.0)
**Tail length**	46.0	45.5 ± 3.6	36.5	89.1 ± 2.6	76.6 ± 3.4	72.7 ± 9.2	49.4 ± 3.3	46.5 ± 1.7
		(36.0–49.5)		(86.0–90.0)	(72.0–80.0)	(62.5–88.5)	(45.5–54.0)	(45.5–48.5)
**J**	10.5	9.6 ± 1.2	8.5	22.4 ± 4.7	24.4 ± 3.2	22.0 ± 2.3	8.0 ± 0.4	10.2 ± 2.1
		(7.0–12.0)		(14.5–26.5)	(22.0–30.0)	(20.0–25.5)	(7.5–8.5)	(8.5–12.5)
**Spicules**	-	-	74.0	-	-	-		
**Lateral accessory piece**	-	-	20.5	-	-	-		

^a^ Measurements are in μm (except for L) and in the form: mean ± standard deviation (range).

^b^ Abbreviations as defined in Jairajpuri & Ahmad [51]. a, body length/maximum body width; b, body length/pharyngeal length; c, body length/tail length; c', tail length/body width at anus; V (distance from anterior end to vulva/body length) x 100; T (distance from cloacal aperture to anterior end of testis/body length) x 100; J (hyaline tail region length).

#### Measurements, morphology and distribution

Morphometric variability is described in Table 6 and morphological traits in Figs 5, 6 and 7. In addition to the type locality, *Xiphinema celtiense* sp. nov. was found in the rhizosphere soil of wild olive (*Olea europaea* subsp. *silvestris* (Miller) Lehr) in one additional locality belonging to Córdoba province. (Table 1, Fig 1).

#### Relationships

**Fig 8:**
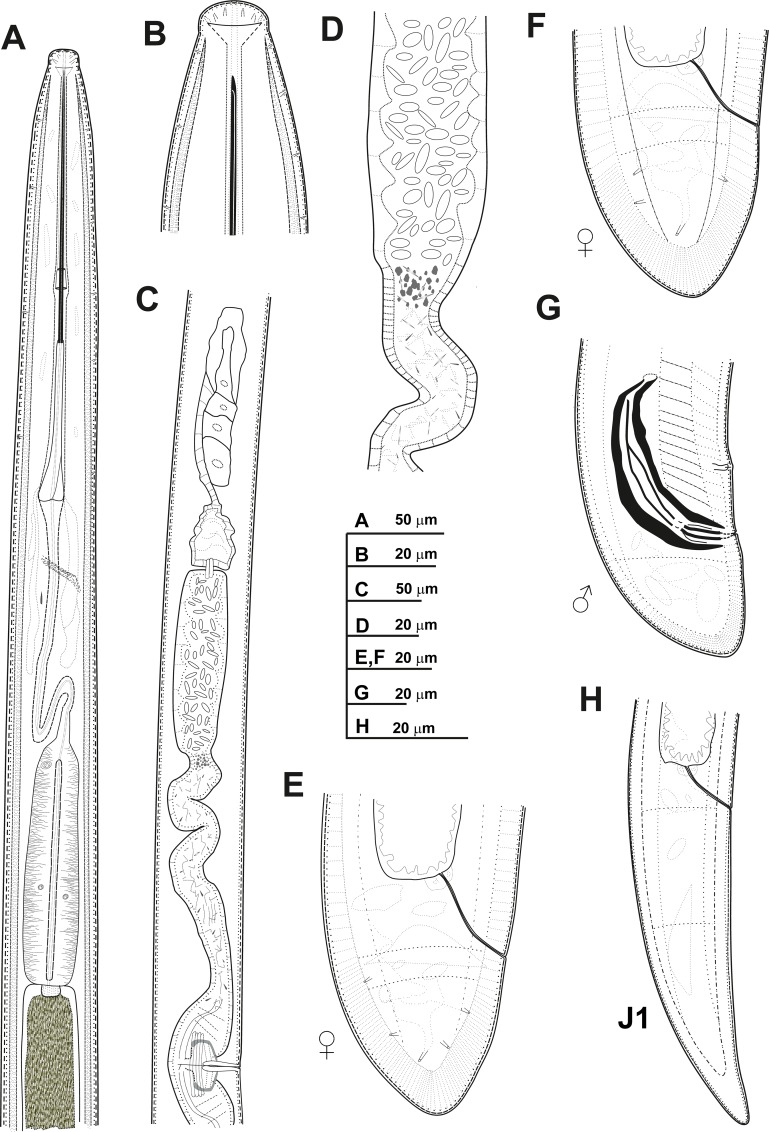
**Line drawings of *Xiphinema iznajarense* sp. nov., female paratypes, male and first-stage juvenile** A) Pharyngeal region. B) Detail of lip region. C) Anterior female genital branch showing Z-differentiation. D) Detail of Z-differentiation. E-F) Female tails. G) Male tail. H) First-stage juvenile tail (J1).

**Fig 9:**
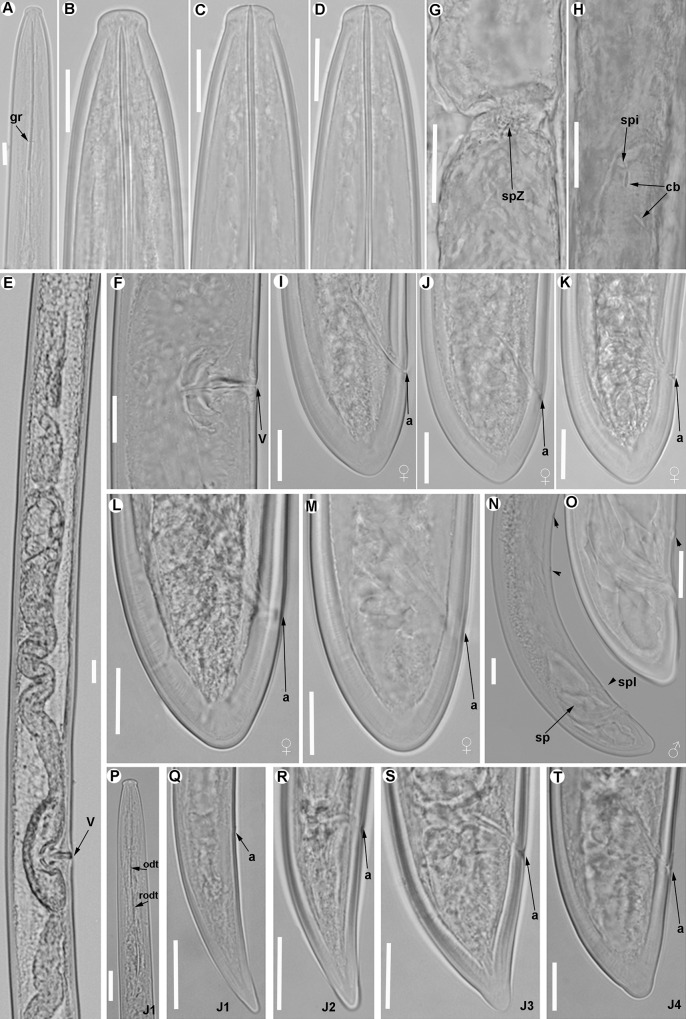
**Light micrographs of *Xiphinema iznajarense* sp. nov., female paratypes, male and juvenile stages** A-D) Female anterior regions. E) Detail of anterior female gonad. F) Vulval region. G-H) Detail of female genital track showing Z-differentiation. I-M) Female tails. N) Male tail with detail of spicules. P) Detail of first-stage anterior region. Q-T) First-, second-, third-, and fourth-stage juvenile (J1-J4) tails, respectively. Abbreviations: a = anus; cb = crystalloid bodies; gr = guiding-ring; odt = odontostyle; rodt = replacement odontostyle; sp = spicules; spi = spiniform structures; spl = ventromedian supplements; spZ = Z-differentiation; v = vulva. Scale bars = 20 μm.

**Fig 10:**
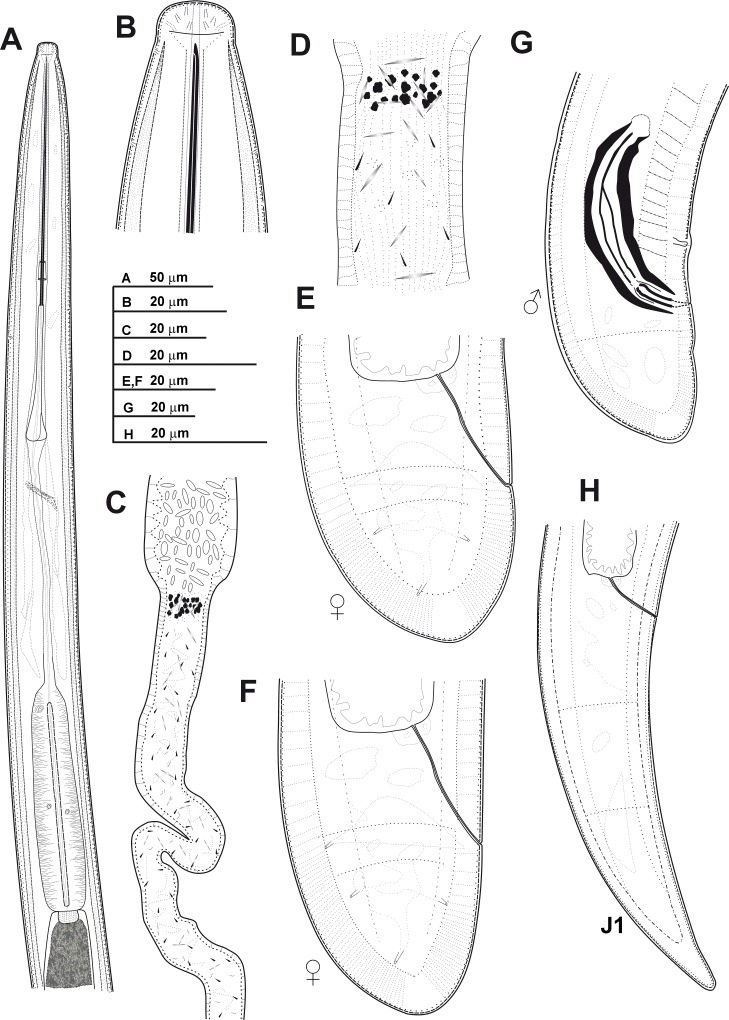
Line drawings of *Xiphinema mengibarense* sp. nov., female paratypes, male and first-stage juvenile. A) Pharyngeal region. B) Detail of lip region. C,D) Detail of Z-differentiation. E,F) Female tails. G) Male tail. H) First-stage juvenile tail (J1).

**Fig 11:**
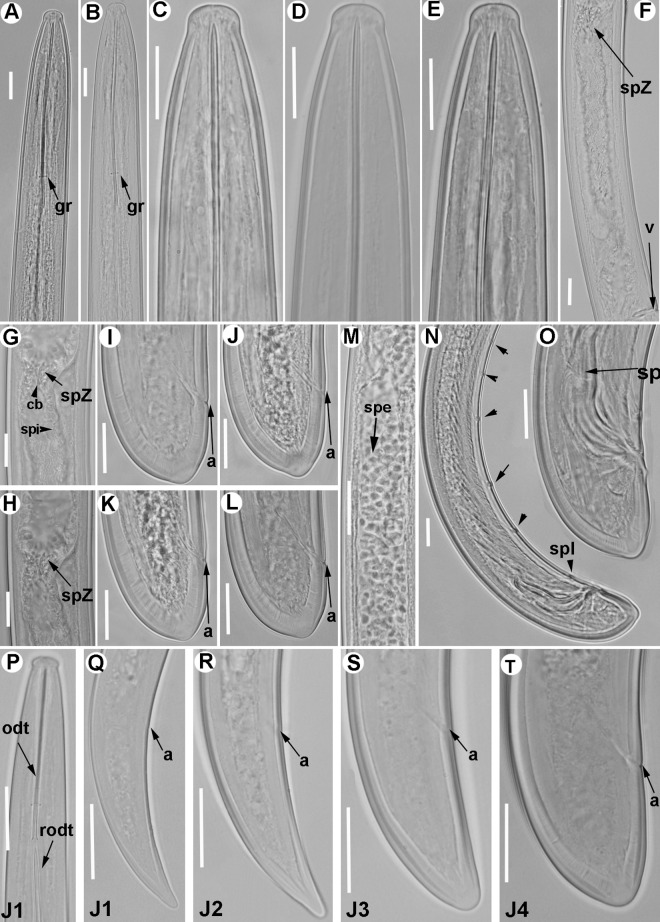
**Light micrographs of *Xiphinema mengibarense* sp. nov., female paratypes, male and juvenile stages** A-E) Female anterior regions. F-H) Detail of female genital track showing Z-differentiation. I-L) Female tails. M) Detail of male genital track showing sperm cells. N-O) Male tail with detail of spicules and ventromedian supplements. P) Detail of first-stage anterior region. Q-T) First-, second-, third-, and fourth-stage juvenile (J1-J4) tails, respectively. Abbreviations: a = anus; cb = crystalloid bodies; gr = guiding-ring; odt = odontostyle; rodt = replacement odontostyle; sp = spicules; spe = sperm cells; spi = spiniform structures; spl = ventromedian supplements; sss = spZ = Z-differentiation; v = vulva. Scale bars = 20 μm.

According to the polytomous key by Loof & Luc [27] and sorting on matrix codes A (type of female genital apparatus), C (tail shape), D (c´ ratio), E (vulva position), F (body length), and G (total spear length (odontostyle + odontophore), *X*. *celtiense* sp. nov. groups with *X*. *iznajarense* sp. nov., *X*. *coronatum* Roca, 1991 [77], and *X*. *turcicum*. Firstly, *X*. *celtiense* sp. nov. can be clearly differentiated from these *Xiphinema* spp. in the absence of spiniform structures in the tubular part of uterus (Figs 8 and 9; [11, 77]. In addition, *X*. *celtiense* sp. nov. mainly differs from *X*. *iznajarense* sp. nov. by slightly lower a and c ratios (64.8–81.0, 97.5–143.9 *vs* 75.2–106.0, 119.4–175.5, respectively), posterior vulva position (50.0–55.0 *vs* 46.0–51.0%), a longer odontostyle and odontophore (145.0–169.0, 89.0–103.0 μm *vs* 132.0–151.0, 80.0–91.5 μm, respectively) resulting in a longer stylet length (241.0–263.05 *vs* 213.0–234.0 μm), a narrower lip region (13.5–16.0 *vs* 15.5–17.0 μm), frequency of males (extremely rare *vs* frequent), and the female and J1 tail shape (Figs 7–10, Tables 6 and 7). On the other hand, *X*. *celtiense* sp. nov. differs from *X*. *coronatum* in having a longer body length (4.7–5.5 *vs* 3.8–4.6 mm), posterior vulva position (50.0–55.0 *vs* 47.1–51.8%), and presence *vs* absence of crystalloid bodies along uterus [77]. Finally, it can be mainly differentiated from *X*. *turcicum* by slightly higher a and c ratios (64.8–81.0, 97.5–143.9 *vs* 52.4–80.3, 83.1–128.0, respectively), presence *vs* absence of crystalloid bodies in the tubular portion of uterus, and different shape of J1 tail (dorsally convex and ventrally concave *vs* dorsally convex and ventrally almost straight) although in both species the tail ends in a knob-like expansion more or less separated from the anterior part of tail (Figs 6 and 7; [11, 72]).

**Table 7:** Morphometrics of females, males and juvenile stages of *Xiphinema iznajarense* sp. nov. from the rhizosphere of cultivated olive at Iznájar (Córdoba province) southern Spain^a^.

Host/locality, sample code	cultivated olive, Iznájar (Córdoba province) JAO25						
Characters/ratios^b^	Holotype	Paratype Females	Paratype Males	J1	J2	J3	J4
**n**		20	9	3	3	6	6
**L (mm)**	4.6	5.3 ± 0.37	5.4 ± 0.34	1.13 ± 0.06	1.74 ± 0.10	2.56 ± 0.18	3.87
		(4.5–5.8)	(4.8–5.8)	(1.07–1.17)	(1.61–1.86)	(2.25–2.77)	
**a**	85.4	89.7 ± 6.8	96.4 ± 9.3	49.3 ± 2.8	58.6 ± 10.3	62.3 ± 10.7	79.7
		(75.2–106.3)	(82.8–110.2)	(47.4–52.5)	(51.8–69.2)	(52.1–80.6)	
**b**	10.5	10.7 ± 0.8	10.0 ± 0.9	4.3 ± 0.5	5.8 ± 10.3	6.7 ± 0.9	8.8
		(9.7–12.8)	(8.4–11.3)	(3.8–4.9)	(5.3–6.5)	(5.3–8.1)	
**c**	121.8	134.9 ± 13.2	136.4 ± 10.3	21.5 ± 2.2	40.3 ± 13.0	56.1 ± 2.8	90.9
		(119.4–175.5)	(122.3–153.7)	(19.2–23.7)	(33.3–51.7)	(51.7–59.1)	
**c´**	0.9	1.0 ± 0.1	0.9 ± 0.01	2.9 ± 0.4	1.9 ± 0.4	1.4 ± 0.2	1.1
		(0.7–1.1)	(0.8–1.0)	(2.5–3.3)	(1.6–2.2)	(1.2–1.6)	
**V or T**	49.0	47.7 ± 1.3	58.5 ± 8.3	-	-	-	-
		(46.0–51.0)	(45.2–69.0)	-	-	-	-
**Odontostyle**	132.0	140.9 ± 4.7	140.0 ± 4.1	62.7 ± 6.8	81.0 ± 4.6	100.3 ± 3.0	126.0
		(132.0–151.0)	(132.0–145.5)	(55.0–68.0)	(78.5–85.0)	(96.0–103.5)	
**Odontophore**	81.0	84.6 ± 3.2	82.1 ± 4.6	49.2 ± 2.5	58.7 ± 1.8	66.3 ± 2.4	76.0
		(80.0–91.5)	(74.0–89.0)	(47.5–52.0)	(57.5–60.5)	(64.0–70.5)	
**Total stylet**	213.0	226.2 ± 5.2	222.1 ± 6.6	-	-	-	-
		(213.0–234.0)	(213.0–230.0)	-	-	-	-
**Replacement odontostyle**	-	-	-	80.8 ± 3.4	100.8 ± 0.4	120.8 ± 4.3	143.0
				(77.0–83.5)	(99.5–103.2)	(115.5–127.0)	
**Lip region diam.**	16.5	16.1 ± 0.4	15.7 ± 0.3	9.8 ± 0.3	11.2 ± 0.7	12.5 ± 0.0	14.0
		(15.5–17.0)	(15.5–16.0)	(9.5–10.0)	(10.5–11.5)	(12.5–12.5)	
**Oral aperture-guiding ring**	120.5	119.5 ± 3.6	121.6 ± 4.5	47.3 ± 2.0	65.0 ± 1.1	84.3 ± 2.3	103.0
		(113.0–125.0)	(117.0–129.0)	(45.5–49.5)	(63.5–66.5)	(81.0–88.0)	
**Tail length**	37.5	39.6 ± 2.8	39.4 ± 2.2	52.8 ± 3.1	50.8 ± 2.5	45.8 ± 2.4	42.5
		(32.5–44.0)	(35.5–42.0)	(49.5–55.5)	(48.5–52.0)	(43.0–48.5)	
**J**	11.5	10.1 ± 2.1	8.8 ± 1.1	11.2 ± 0.6	11.2 ± 3.2	10.9 ± 2.5	10.0
		(7.0–14.0)	(7.0–10.5)	(10.5–11.5)	(9.0–14.5)	(8.0–13.5)	
**Spicules**	-	-	70.7 ± 2.8	-	-	-	-
			(66.0–75.5)				
**Lateral accessory piece**	-	-	14.9 ± 0.9	-	-	-	-
			(13.5–16.0)				

^a^ Measurements are in μm (except for L) and in the form: mean ± standard deviation (range).

^b^ Abbreviations as defined in Jairajpuri & Ahmad [51]. a, body length/maximum body width; b, body length/pharyngeal length; c, body length/tail length; c', tail length/body width at anus; V (distance from anterior end to vulva/body length) x 100; T (distance from cloacal aperture to anterior end of testis/body length) x 100; J (hyaline tail region length).

In addition, *X*. *celtiense* sp. nov. is molecularly related to *X*. *hispanum* Lamberti, Castillo, Gómez Barcina & Agostinelli, 1992 [65] *and X*. *cohni* Lamberti, Castillo, Gómez Barcina & Agostinelli, 1992 [65], but it can be clearly differentiated by a combination of characters discussed below. From *X*. *hispanum* it mainly differs in having a longer odontostyle (145.0–169.0 *vs* 131.2–142.3 μm), and female tail shape (hemispherical *vs* widely conical or dorso-ventrally convex) [11, 65]. And from *X*. *cohni* it mainly differs by the presence *vs* absence of Z-differentiation containing numerous granular bodies, and female tail shape (hemispherical *vs* convex-conoid or conical ending in a terminal bulge (Figs 6 and 7; [17, 65]).

#### Molecular divergence of the new species

D2-D3 sequences from *X*. *celtiense* sp. nov. (KX244889-KX244890) differed with the closest related species, *X*. *hispanum* (GU725074) by 24 nucleotides and 3 gaps (97% similarity) and from *X*. *cohni* (KC567173, KX244901) from 27 nucleotides and 1 indel (97% similarity). Intraspecific variation of D2-D3 segments detected between the two studied population of *X*. *celtiense* sp. nov. consisted of 7 nucleotides (99% similarity), and no indels (Table 5). ITS1 (KX244926-KX244927) also showed some similarity, 87% (136 nucleotides and 28 indels) with *X*. *hispanum* (GU725061) and 86% (141 nucleotides and 34 indels) with *X*. *cohni* (KX244933). Intraspecific variation of the ITS1 for these sequences (KX244926-KX244927) was 44 nucleotides and 18 gaps, 95% similarity (Table 5). Some microsatellites were found in these sequences contributing to sequence variation. Finally, the partial 18S of *X*. *celtiense* sp. nov. (KX244943) showed a high level of similarity (99%) with several sequences deposited in GenBank such as *X*. *hispanum* (GU725083), *X*. *adenohystherum* (GY725084), and *X*. *nuragicum* (GU725080).

***Xiphinema iznajarense* Archidona-Yuste, Navas-Cortés, Cantalapiedra-Navarrete, Palomares-Rius & Castillo, sp. nov.** *urn*:*lsid*:*zoobank*.*org*:*act*:*4B6E1D31-033F-41C4-A7D0-1F60E4945F35*

Figs 5, 8 and 9.

#### Holotype

Adult female, collected from the rhizosphere of cultivated olive (*Olea europaea* subsp. *europaea* L.) (37°15'39.4"N, 004°19'20.02"W), at Iznájar, Córdoba province, Spain; collected by J.E. Palomares-Rius, December 3, 2014; mounted in pure glycerine and deposited in the nematode collection at Institute for Sustainable Agriculture (IAS) of Spanish National Research Council (CSIC), Córdoba, Spain (collection number JAO-25-1).

#### Paratypes

Female, male and juvenile paratypes extracted from soil samples collected from the same locality as the holotype; mounted in pure glycerine and deposited in the following nematode collections: Institute for Sustainable Agriculture (IAS) of Spanish National Research Council (CSIC), Córdoba, Spain (collection numbers JAO-25-2-JAO-25-7); one female and one male at Istituto per la Protezione Sostenibile delle Piante (IPSP), Consiglio Nazionale delle Ricerche (CNR), Bari, Italy (JAO-25-12); two females and one juvenile at Royal Belgian Institute of Natural Sciences, Brussels, Belgium (RIT 853); and two females, one male, and one juvenile at USDA Nematode Collection, Beltsville, MD, USA (T-6777p); collected by J.E. Palomares-Rius, December 3, 2014.

#### Diagnosis

*Xiphinema iznajarense* sp. nov. is an amphimictic species belonging to morphospecies Group 5 from *X*. non-*americanum*-group species *sensu* Loof & Luc [27]. It is characterized by a moderately long body (4.5–5.8 mm), assuming an open C-shaped when heat-relaxed; lip region frontally rounded and almost laterally straight, usually low and distinctly set off from body contour, 15.5–17.0 μm wide; moderately long odontostyle (132.0–151.0 μm); vulva position slightly anterior to mid body; reproductive system didelphic-amphidelphic with both branches about equally developed, Z-differentiation containing small and numerous granular bodies, uterus tripartite with small crystalloid bodies in higher number than small spiniform structures, and presence of prominent wrinkles from the uterine wall; female tail short and conoid, dorso-ventrally convex, ending in a rounded terminus and bearing four to five pairs of caudal pores; c´ ratio (0.7–1.1); males frequent with long spicules (*ca* 71 μm), and one pair of adanal supplement plus 4–5 pairs of ventromedian supplements; and specific D2-D3, ITS1 rRNA and partial 18S rRNA sequences (GenBank accession numbers KX244891-KX244892, KX244928-KX244929, and KX244944, respectively). According to the polytomous key of Loof & Luc [27], the new species has the following specific alphanumeric codes (codes in parentheses are exceptions): A4-B2+3-C7-D6(5)-E5(6)-F5-G3-H2-I3-J6-K2-I2.

#### Etymology

The species epithet refers to the type locality, Iznájar, where the species was detected.

#### Description of taxa. Female

Habitus in specimens killed by gentle heat usually almost straight anterior to the vulva, more curved behind the vulva, occasionally open C-shaped. Cuticle 2.0–4.0 μm thick at mid-body, more thickened in the lip region (4.0–6.0 0 μm wide) and tail tip region (5.5–10.0 μm wide). Lateral hypodermical chords occupying about 26–46% of the corresponding maximum body diameter. Lip region hemispherical, broadly rounded frontally, usually low and offset from body contour by a shallow constriction; 15.5–17.0 μm wide and 5.5–7.5 μm high. Amphidial fovea aperture extending for *ca* 63–74% of lip region diam. and located at *ca* two-thirds of lip region height. Guiding ring with average guiding sheath length of 12.0 μm. Odontostyle moderately long, 1.5–1.8 times longer than odontophore, and the latter with well-developed flanges in the most of specimens studied, 11.5–22.0 μm wide. Pharynx consisting of an anterior slender narrow part 265–414 μm long, extending to a cylindrical, terminal pharyngeal bulb occupying *ca* 23–36% of total pharyngeal length, cylindrical, 117–153 μm long and 20–29 μm wide. Glandularium 101–135 μm long. Nucleus of dorsal pharyngeal gland (DN) located at beginning of basal bulb (11.6–12.6%), ventrosublateral nuclei (SVN) situated *ca* halfway along bulb (50.5–57.8%) (position of gland nuclei calculated as described by Loof & Coomans [74]. In some specimens studied, vestigium (tip of reserve odontostyle), 2.5 μm long, observed in anterior region of slender part of pharynx. Cardia conoid and variable in length, 11.5–22.0 μm long. Prerectum reaching about 10–15% of nematode body from the anus to anterior part. Rectum 29.5–38.0 μm long ending in anus as a small rounded slit. Reproductive system didelphic-amphidelphic with branches about equally developed. Each branch composed of a short ovary (63.5–122.0 *μ*m long), a reflexed oviduct with well-developed *pars dilatata oviductus* separated from uterus by a well-developed sphincter. Uteri tripartite, comprising a developed *pars dilatata uteri* continuing into a narrower, muscular tube-like portion, and a well-developed Z-differentiation with weakly muscularised wall and containing numerous small granular bodies. Uterine wall wrinkles present along uterus, being more numerous in the proximal part of *pars dilatata uteri* and ovejector (Fig 9E). Small spiniform structures and crystalloid bodies present, in low number, along uterus and observed when tubular part of uterus is wider and without wrinkles (Figs 8 and 9G and 9H). In some specimens studied and in a proximal part of *pars dilatata uteri*, spindle-shaped sperm cells were observed, being variable in length (3.0–6.5 μm long). Ovejector well-developed 35.5–56.0 μm wide, vagina perpendicular to body axis, 18.0–24.0 μm long in lateral view. Vulva slit-like, pre-equatorial. Tail conoid and short, dorso-ventrally convex, ending in a rounded and broadly terminus, bearing in four to five pairs of caudal pores on each side.

#### Male

Frequent but less abundant than female (ratio = 1: 2). Morphologically similar to female except for genital system and more curved posterior part of body. Male genital tract diorchic with testes containing multiple rows of different stages of spermatogonia. Tail short, convex-conoid with short bulge rounded terminus and thickened outer cuticular layer (Figs 8, 9N and 9O). Spicules moderately long and slightly curved ventrally; lateral guiding pieces more or less straight or with curved proximal end. One pair of adanal and 4–5 mid-ventral supplements.

#### Juveniles

All four juvenile stages (first-, second-, third- and fourth-stage) were identified using morphological characters such as body length, length of replacement and functional odontostyle (Table 7, Fig 5) [75, 76]. In particular, J1 were characterised by position of replacement odontostyle just posterior to functional odontostyle, its tip touching or very close to base of functional odontostyle; tail blunty conoid elongate with a c´ ratio ≥ 3.8 (Figs 8 and 9Q); and odontostyle length *ca* 63 μm. Tail morphology in second-stage juvenile similar to J1 expect for the presence a slightly depression at the level of the hyaline region in both sides. On the other hand, the tail was conoid and subdigitate with a rounded terminus for J3, while for fourth-stage juvenile was conoid with rounded terminus and short bulge (Fig 9T). All juvenile developmental stages showed a tail becoming progressively shorter and stouter in each moult, and shorter distance from anterior end to guiding-ring in each moult (Table 7, Fig 9Q–9T).

#### Measurements, morphology and distribution

Morphometric variability is described in Table 7 and morphological traits in Figs 5, 8 and 9. *Xiphinema iznajarense* sp. nov. was only found in type locality, Iznájar (Córdoba province), being extracted from the rhizosphere of cultivated olive (*Olea europaea* subsp. *europaea* L.) (Table 1, Fig 1).

#### Relationships

According to the polytomous key by Loof & Luc [27] and sorting on matrix codes A (type of female genital apparatus), C (tail shape), D (c´ ratio), E (vulva position), F (body length), and G (total spear length (odontostyle + odontophore), *X*. *iznajarense* sp. nov. closely resembles with *X*. *celtiense* sp. nov., *X*. *coronatum* and *X*. *turcicum*. *Xiphinema iznajarense* sp. nov. can be differentiated from *X*. *celtiense* sp. nov. by the characters discussed above. From *X*. *coronatum* it differs in having a longer body (4.5–5.8 *vs* 3.8–4.6 mm), higher a ratio (75.2–106.3 *vs* 65.5–75.5), a shorter odontophore and lower oral aperture-guiding ring distance (80.0–91.5, 113.0–125.0 μm *vs* 90.0–101.2, 142.3–154.1 μm, respectively), frequency of males (frequent *vs* extremely rare), presence *vs* absence of crystalloid bodies in the tubular portion of uterus, female tail shape (widely conical *vs* hemispherical), and shape of J1 tail (conoid elongate with rounded terminus *vs* a long clavate peg) (Figs 8 and 9; [77]). Finally, *X*. *iznajarense* sp. nov. can be differentiated from *X*. *turcicum* by slightly higher a and c ratios (75.2–106.3, 119.4–175.5 *vs* 52.4–80.3, 83.1–128.3, respectively), a shorter odontostyle length (132.0–151.0 *vs* 152.0–182.0 μm), the presence *vs* absence of crystalloid bodies along uterus, the frequency of males (frequent *vs* rare), the female tail shape (widely conical *vs* hemispherical), and shape of J1 tail (conoid elongate *vs* dorsally convex and ventrally almost straight ending in a knob-like expansion more or less separated from the anterior part of tail) (Figs 8 and 9; [11, 72]).

In addition, *X*. *iznajarense* sp. nov. is molecularly related to *X*. *hispidum* Roca & Bravo, 1994 [78] and *X*. *adenohystherum*, but it can be clearly differentiated by a combination of characters discussed below. From *X*. *hispidum* it can be differentiated by higher c ratio (119.4–175.5 *vs* 70.1–96.5), lower c´ ratio (0.7–1.1 *vs* 1.4–2.2), a longer odontostyle (132.0–151.0 *vs* 107.0–131.0 μm), and the presence *vs* absence of crystalloid bodies along uterus (Figs 8 and 9; [31, 78]). And from *X*. *adenohystherum* it clearly differs in having the presence *vs* absence of Z-differentiation containing numerous granular bodies, and presence *vs* absence of crystalloid bodies in the tubular portion of uterus (Figs 8 and 9; [17, 65]).

#### Molecular divergence of the new species

D2-D3 region of *X*. *iznajarense* sp. nov. (KX244891-KX244892) was 97% similar (26 nucleotides and 1 indel) to *X*. *adenohystherum* (GU725075), *X*. *hispidum* (KC567181) and 95% similar (36 nucleotides and 2 indels) to *X*. *hispanum* (GU725074). No intraspecific variation of D2-D3 segments was detected amongst the studied individuals (100% similarity) (Table 5). Similarly, ITS1 (KX244928-KX244929) also showed some similarity with *X*. *hispanum* (GU725061), *X*. *adenohystherum* (GU725063) and *X*. *hispidum* (HM921367) with similarity values of 88% (131 nucleotides and 31 indels), 87% (145 nucleotides and 29 indels) and 84% (175 nucleotides and 52 indels), respectively (Table 5). ITS1 also showed a low intraspecific variation between the studied individuals, 9 nucleotides and no indels. The partial 18S of *X*. *iznajarense* sp. nov. (KX244944) closely matched with several species of *Xiphinema*, some of them were *X*. *adenohystherum* (GU725084), *X*. *hispanum* (GU725083), *X*. *gersoni* Roca & Bravo, 1993 [79] (KC567154) and *X*. *sphaerocephalum* Lamberti, Castillo, Gómez Barcina & Agostinelli, 1992 [65] (GU725082).

***Xiphinema mengibarense* Archidona-Yuste, Navas-Cortés, Cantalapiedra-Navarrete, Palomares-Rius & Castillo, sp. nov.** urn:lsid:zoobank.org:act:C42E7495-B8AD-42EF-BB3C-3F0E34476F2C

Figs 5, 10 and 11

#### Holotype

Adult female, collected from the rhizosphere of cultivated olive (*Olea europaea* subsp. *europaea* L.) (38°01'21.72"N, 003°46'38.68"W), at Mengíbar, Jaén province, Spain; collected by J. Martín-Barbarroja, March 25, 2012; mounted in pure glycerine and deposited in the nematode collection at Institute for Sustainable Agriculture (IAS) of Spanish National Research Council (CSIC), Córdoba, Spain (collection number O3C4-01).

#### Paratypes

Female, male and juvenile paratypes extracted from soil samples collected from the same locality as the holotype; mounted in pure glycerine and deposited in the following nematode collections: Institute for Sustainable Agriculture (IAS) of Spanish National Research Council (CSIC), Córdoba, Spain (collection numbers O3C4-02-O3C4-08); one female, one male and one juvenile at Istituto per la Protezione Sostenibile delle Piante (IPSP), Consiglio Nazionale delle Ricerche (CNR), Bari, Italy (O3C4-19); one female and one male at Royal Belgian Institute of Natural Sciences, Brussels, Belgium (RIT 854); and one female and one male at USDA Nematode Collection, Beltsville, MD, USA (T-6776p); collected by J. Martín-Barbarroja, March 25, 2012.

#### Diagnosis

*Xiphinema mengibarense* sp. nov. belongs to the *X*. non-*americanum* Group 5 in Loof and Luc [27]; and it is characterized by a moderate long body (3.8–4.8 mm), assuming an open C-shaped when heat-relaxed; lip region anteriorly rounded set off from body contour by a slightly depression, 12.5–15.5 μm wide and 5.5–8.5 μm high; guiding-ring located 104–122 μm from anterior end; moderately long odontostyle and odontophore (120.0–131.5, 73.0–83.5 μm, respectively); vulva slightly posterior to mid body; reproductive system didelphic-amphidelphic with both branches about equally developed including a Z-differentiation with muscularised wall and containing about 8–15 small granular bodies, uteri tripartite full of spindle shaped sperm in some specimens, and very small spiniform structures and crystalloid bodies in low number that in some specimens they can be confused with the wrinkles of the uterine wall; female tail broadly dorsally convex-conoid with rounded terminus, a short bulge, and a distinct terminal blind canal; c’ ratio (0.7–1.1); males frequent but less abundant than females, with spicules 57.5–66.0 μm long and 5 to 6 ventromedian supplements; and specific D2-D3, ITS1 rRNA and partial 18S rRNA sequences (GenBank accession numbers KX244893-KX244895, KX244930-KX244931, and KX244945, respectively). According to the polytomous key of Loof & Luc [27], the species belongs to *Xiphinema* non-americanum Group 5 and has the following specific alphanumeric codes (codes in parentheses are exceptions): A4-B2+3-C5a-D6(5)-E6(5)-F45-G32-H2-I2-J6-K2-I2.

#### Etymology

The species epithet refers to the type locality, Mengíbar, where the species was detected.

#### Description of taxa. Female

Body cylindrical in an open C-shape when heat relaxed. Cuticle 3.1 ± 0.3 (2.0–4.5) μm thick at post-lip region, 2.8 ± 0.5 (2.0–4.0) μm wide at mid-body, but thicker just posterior to anus, 6.4 ± 1.8 (5.0–10.0) μm thick. Lateral chord 13.0 ± 4.8 (8.0–20.0) μm wide, occupying 17–42% of corresponding body diam. Lip region flatly rounded, slightly offset from body contour by a depression, 13.9 ± 0.7 (12.5–15.5) μm wide and 6.9 ± 0.8 (5.5–8.5) μm high. Amphidial fovea aperture extending for *ca* 64–78% of lip region diam. and located at *ca* two-thirds of lip region height. Guiding ring and guiding sheath variable in length depending on degree of protraction/retraction of stylet. Odontostyle moderately long, 1.5–1.7 times longer than odontophore, in the most specimens the latter with moderate-developed flanges, but in some specimens it was observed weaker, 8.5–14.0 μm wide. Pharynx composed by an anterior slender narrow flexible part 317–417 μm long, and a posterior muscular expanded part with three nuclei. Terminal pharyngeal bulb variable in length, 120–173 μm long and 19.5–29.5 μm wide. Glandularium 104–148 μm long. Nucleus of dorsal pharyngeal gland (DN) located at beginning of basal bulb (9.2–15.0%), ventrosublateral nuclei (SVN) situated *ca* halfway along bulb (45.7–58.0%) (position of gland nuclei calculated as described by Loof & Coomans [74]). Cardia conoid, 10.4 ± 0.8 (8.0–12.5) μm long. Prerectum variable in length 586.2 ± 93.2 (444.0–772.0) μm long, or occupying 10–18% of body length. Rectum 18.5–36.0 μm long ending in anus as a small rounded slit. Reproductive system didelphic-amphidelphic with both branches about equally developed. Each branch composed of short reflexed ovary 65–97 μm long and a largely tubular oviduct with enlarged *pars dilatata oviduct* separated from uterus by a well-developed sphincter. Uteri tripartite, comprising a well-developed *pars dilatata uteri* continuing into a narrower, muscular tube-like portion including a Z-differentiation with weakly muscularised wall and containing 8–15 small granular bodies. Wrinkles in uterine wall present, being more numerous in proximal part of *pars dilatata uteri*. Uteri and proximal part of *pars dilatata uteri* often with abundant spindle shaped sperm cells, 2.0–8.0 μm long. In some specimens, and when devoid of sperm, low numbers of small spiniform structures and crystalloid bodies seen along uterus, being more abundant about at Z-differentiation level. Ovejector well-developed, 36–47 μm wide, vagina perpendicular to body axis, 16.5–23.0 μm long or 34–47% of corresponding body diam. in lateral view. Vulva slit-like, situated in mid-body region. Tail broadly dorsally convex-conoid (slightly concave ventrally and hemispherical dorsally), with slightly bulging rounded terminus with a distinct terminal blind canal. Three to four caudal pores present on each side.

#### Male

Functional, less abundant than females (ratio = 1: 2). Reproductive system diorchic with testes occupying 45–57% of body length, and spindle-shaped sperm. Spicules dorylaimoid, massive, well sclerotised, 57.5–66.0 μm long, ventrally curved with tubular lateral guiding pieces 13.5–18.0 μm long. One pair of adanal supplements located at 16.6 ± 1.2 (15.5–19.0) μm from cloacal opening and a series of four to five ventromedian supplements. Tail similar to that of female, dorsally more convex than female, and ending in a rounded terminus with short bulge.

#### Juveniles

All four juvenile stages were found and detected using body length, length of replacement and functional odontostyle (Table 8, Figs 5 and 11) [75, 76]. J1 were characterised by position of replacement odontostyle just posterior to functional odontostyle, its tip touching or very close to base of functional odontostyle; tail elongate, dorsally convex and ventrally concave with a slightly dorsal depression at hyaline region with a c’ ratio ≥ 3.1 (Figs 10 and 11Q); odontostyle length *ca* 53 μm, and shorter distance from anterior end to stylet guiding-ring than that in adult stages. Tail morphology in second and third juvenile stages similar to J1, becoming progressively shorter and stouter in each progressively moult. However, tail shape in fourth-stage similar into that of female, broadly dorsally convex-conoid with slightly bulging rounded terminus (Fig 11Q and 11T).

**Table 8:** Morphometrics of females, males and juvenile stages of *Xiphinema mengibarense* sp. nov. from the rhizosphere of cultivated olive at several localities (Jaén province) southern Spain^a^.

Host/locality, sample code	cultivated olive, Mengíbar (Jaén province) O3V4						
Characters/ratios^b^	Holotype	Paratype Females	Paratype Males	J1	J2	J3	J4
**n**		20	11	5	6	6	6
**L (mm)**	4.6	4.3 ± 0.25	4.2 ± 0.28	1.24 ± 0.62	1.82 ± 0.96	2.40 ± 0.93	3.27 ± 0.44
		(3.8–4.8)	(3.6–4.6)	(1.18–1.34)	(1.71–1.89)	(2.32–2.57)	(3.21–3.34)
**a**	95.1	88.4 ± 5.2	94.0 ± 7.6	54.5 ± 1.5	66.8 ± 5.0	76.9 ± 9.1	80.7 ± 6.2
		(80.0–98.2)	(83.6–109.3)	(53.2–57.0)	(61.8–74.5)	(65.4–85.1)	(70.4–89.0)
**b**	9.2	8.5 ± 0.7	8.8 ± 0.8	5.7 ± 1.2	6.1 ± 0.7	6.6 ± 0.5	7.3 ± 0.3
		(6.5–9.8)	(7.3–10.2)	(4.8–7.7)	(5.4–7.3)	(6.2–7.6)	(6.9–7.7)
**c**	156.4	135.2 ± 13.7	121.0 ± 10.1	24.4 ± 2.1	38.7 ± 1.3	58.2 ± 2.0	91.0 ± 2.6
		(106.0–158.3)	(105.4–135.7)	(21.8–26.5)	(35.3–41.2)	(54.7–60.4)	(87.8–95.6)
**c´**	0.8	0.9 ± 0.1	1.0 ± 0.1	3.4 ± 0.4	2.3 ± 0.1	1.7 ± 0.1	1.1 ± 0.04
		(0.7–1.1)	(0.9–1.2)	(3.1–4.0)	(2.2–2.4)	(1.5–1.8)	(1.0–1.1)
**V or T**	54.0	52.1 ± 1.9	52.0 ± 3.1	-	-	-	-
		(48.5–57.0)	(45.7–57.2)	-	-	-	-
**Odontostyle**	126.5	125.0 ± 3.1	124.4 ± 4.3	52.8 ± 2.8	66.0 ± 1.4	84.9 ± 2.2	105.5 ± 2.8
		(120.0–131.5)	(117.0–131.5)	(48.5–56.0)	(63.5–69.0)	(83.0–88.5)	(101.5–110.0)
**Odontophore**	73.5	76.1 ± 2.5	72.0 ± 1.7	37.3 ± 3.6	46.4 ± 1.1	56.9 ± 1.7	65.5 ± 1.8
		(73.0–83.5)	(69.5–75.5)	(41.0–49.0)	(35.5–51.0)	(55.0–59.5)	(63.5–68.5)
**Total stylet**	200.0	201.1 ± 4.5	196.4 ± 4.3	-	-	-	-
		(194.5–215.0)	(188.0–203.0)	-	-	-	-
**Replacement odontostyle**	-	-	-	64.9 ± 0.9	87.0 ± 2.5	105.0 ± 3.9	127.8 ± 3.5
	-	-	-	(64.0–66.0)	(83.0–91.5)	(99.0–109.5)	(123.5–132.0)
**Lip region diam.**	14.5	13.9 ± 0.7	13.7 ± 0.5	8.7 ± 0.6	9.6 ± 0.4	10.6 ± 2.0	12.1 ± 0.2
		(12.5–15.5)	(12.5–14.0)	(8.0–9.5)	(8.5–10.0)	(10.0–11.5)	(12.0–12.5)
**Oral aperture-guiding ring**	114.0	112.1 ± 5.4	110.5 ± 4.3	44.2 ± 3.6	58.1 ± 0.4	75.3 ± 3.0	91.9 ± 4.2
		(104.0–122.0)	(104.0–118.5)	(41.0–49.0)	(57.0–61.0)	(71.0–79.0)	(87.5–99.5)
**Tail length**	29.5	32.1 ± 3.9	34.8 ± 1.8	50.9 ± 3.2	47.2 ± 3.9	41.3 ± 2.0	35.9 ± 1.0
		(27.0–42.0)	(32.5–38.5)	(47.5–55.5)	(42.5–53.5)	(39.5–44.5)	(34.0–36.5)
**J**	7.5	8.9 ± 1.2	7.9 ± 0.5	8.4 ± 1.2	8.7 ± 1.4	6.8 ± 0.8	7.9 ± 1.3
		(7.5–11.5)	(7.5–9.0)	(7.5–10.0)	(6.5–11.5)	(6.0–7.5)	(6.5–9.5)
**Spicules**	-	-	60.7 ± 2.6	-	-	-	-
			(57.5–66.0)	-	-	-	-
**Lateral accessory piece**	-	-	15.5 ± 1.6	-	-	-	-
	-	-	(13.5–18.0)	-	-	-	-

^a^ Measurements are in μm (except for L) and in the form: mean ± standard deviation (range).

^b^ Abbreviations as defined in Jairajpuri & Ahmad [51]. a, body length/maximum body width; b, body length/pharyngeal length; c, body length/tail length; c', tail length/body width at anus; V (distance from anterior end to vulva/body length) x 100; T (distance from cloacal aperture to anterior end of testis/body length) x 100; J (hyaline tail region length).

#### Measurements, morphology and distribution

Morphometric variability is described in Table 8 and morphological traits in Figs 5, 10 and 11. *Xiphinema mengibarense* sp. nov. was only found in type locality, Mengíbar (Jaén province), being extracted from the rhizosphere of cultivated olive (*Olea europaea* subsp. *europaea* L.) (Table 1, Fig 1).

#### Relationships

According to the polytomous key by Loof & Luc [27] and sorting on matrix codes A (type of female genital apparatus), C (tail shape), D (c´ ratio), E (vulva position), F (body length), and G (total spear length (odontostyle + odontophore), *X*. *mengibarense* sp. nov. groups with *X*. *herakliense* Tzortzakakis et al., 2015 [80], *X*. *hispanum*, and *X*. *lanceolatum* Roca & Bravo, 1993 [81]. Firstly, *Xiphinema mengibarense* sp. nov. can be differentiated from *X*. *herakliense* by higher a and c ratios (80.0–98.2, 106.0–158.3*vs* 59.0–75.0, 83.0–122.0, respectively), a shorter odontostyle (120.0–131.5 *vs* 127.0–157.0 μm), shorter spicules (57.5–66.0 *vs* 70.0–81.0 μm) [80]. On the other hand, *X*. *mengibarense* sp. nov. mainly differs from *X*. *hispanum* in having higher a ratio (80.0–98.2 *vs* 73.1–83.9), a shorter odontostyle (120.0–131.5 *vs* 131.2–142.3 μm), the number of spiniform structures present in the Z-differentiation (lower *vs* abundant), the presence *vs* absence of crystalloid bodies in the tubular portion of uterus, and the frequency of males (frequent *vs* rare) (Figs 10 and 11; [11, 65]). Finally, *X*. *mengibarense* sp. nov. can be differentiated from *X*. *lanceolatum* by higher a ratio (80.0–98.2 *vs* 50.5–75.5), a shorter odontostyle and odontophore (120.0–131.5, 73.0–83.5 μm *vs* 165.5–185.5, 90.0–98.0 μm, respectively) resulting in a shorter stylet (194.5–215.0 *vs* 255.5–283.0 μm), a slightly narrower lip region (12.5–15.5 *vs* 14.5–18.0 μm), posterior vulva position (48.5–57.0 *vs* 43.5–50.0%), the presence *vs* absence of males, and the number of spiniform structures and crystalloid bodies (lower *vs* very abundant) (Figs 10 and 11; [81]).

#### Molecular divergence of the new species

D2-D3 region of *X*. *mengibarense* sp. nov. (KX244893-KX244895) was 94% similar to several *Xiphinema* species such as *X*. *italiae* (HM921351, 48 nucleotides and 12 indels), *X*. *pyrenaicum* Dalmasso, 1969 [82] (GU725073, 46 nucleotides and 15 indels) and *X*. *sphaerocephalum* (GU725076, 48 nucleotides and 10 indels). *Xiphinema mengibarense* sp. nov. showed a high homogeneity for the D2-D3 region (99% similarity, 2 nucleotides) in the three sampled populations (Table 5). The closest species in relation to ITS1 region were *X*. *hispanum* (GU725061) and *X*. *cohni* (KC567159), with a similarity of 84% (183 and 194 nucleotides and 55 and 65 indels, respectively) (Table 5). Low intraspecific variation for the ITS1 region (KX244930-KX244931) was detected among the studied population, 8 nucleotides and no indels. Finally, the partial 18S of *X*. *mengibarense* sp. nov. (KX244945) closely matched (99% similarity) those for *X*. *italiae* (FJ713154), *X*. *pyrenaicum* (GU725085) and *X*. *gersoni* (KC567154).

### Morphology and morphometrics of known *Xiphinema* species

**Fig 12:**
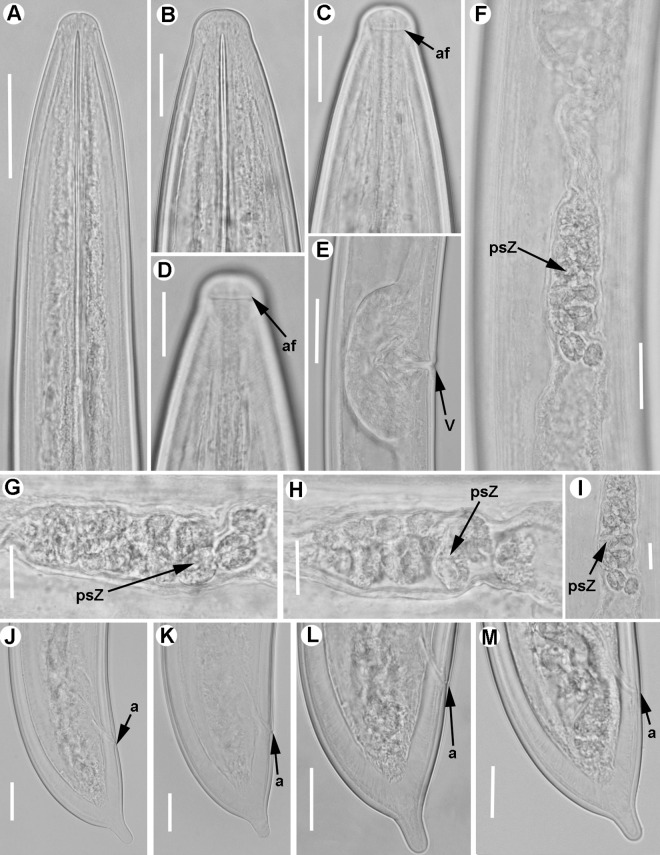
**Light micrographs of *Xiphinema cadavalense* Bravo & Roca, 1995 females from Spain** A) Neck region. B-D) Female lip regions. E) Vulval region. F-I) Details of pseudo-Z organ. J-M) Female tails. Abbreviations: a = anus; af = amphidial fovea; psZ = pseudo-Z organ. Scale bars = 20 μm.

**Fig 13:**
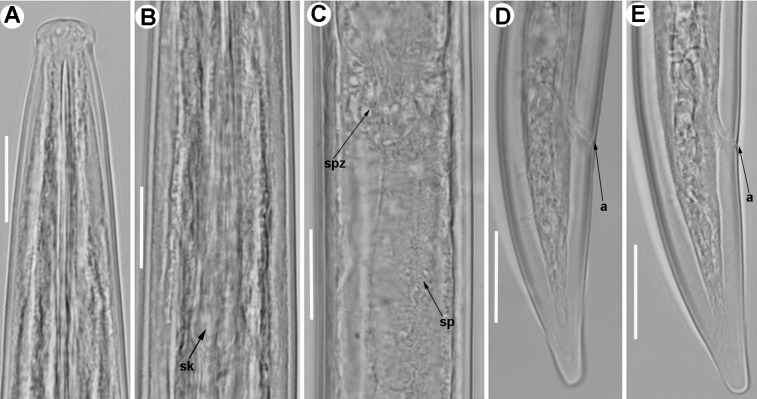
**Light micrographs of *Xiphinema conurum* Siddiqi, 1964 females from Spain** A) Female lip region. B) Female anterior region showing detail of odontophore and flanges. C) Detail of female genital track showing Z-differentiation. D-E) Female tails. Abbreviations: a = anus; sk = flanges; sp = spiniform structures; spZ = Z-differentiation. Scale bars = 20 μm.

**Fig 14:**
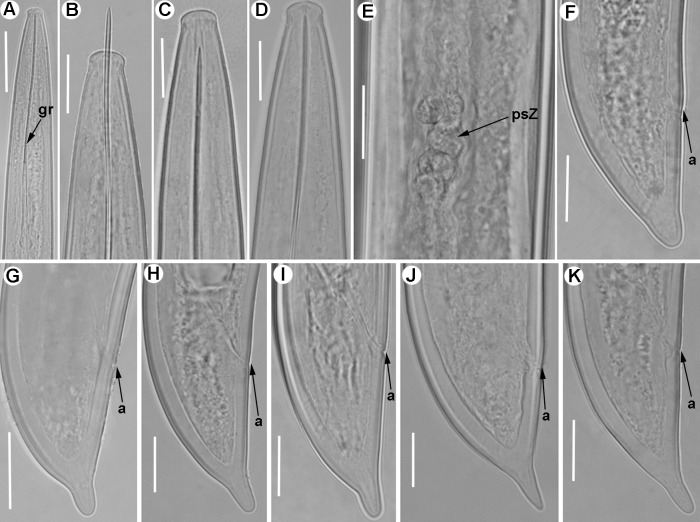
**Light micrographs of *Xiphinema pseudocoxi* Sturhan, 1984, females from Spain** A) Neck region. B-D) Details of lip region. E) Detail of pseudo-Z organ. F-K) Female tails showing morphological variability. Abbreviations: a = anus; gr = guiding ring; psZ = pseudo-Z organ. Scale bars A = 50 μm; B-K = 20 μm.

Morphological and morphometrical data, and molecular delineation (rDNA) of *X*. *adenohystherum*, *X*. *baetica*, *X*. *cohni*, *X*. *coxi europaeum*, *X*. *duriense*, *X*. *hispanum*, *X*. *hispidum*, *X*. *incertum*, *X*. *index* Thorne & Allen, 1950 [83], *X*. *italiae*, *X*. *lupini* Roca & Pereira, 1993 [84], *X*. *macrodora*, *X*. *madeirense* Brown, Faria, Lamberti, Halbrendt, Agostinelli & Jones, 1993 [85], *X*. *nuragicum*, *X*. *oleae*, *X*. *opisthohysterum* Siddiqi, 1961 [86], *X*. *pachtaicum*, *X*. *parapachydermum*, *X*. *plesiopachtaicum*, *X*. *rivesi* Dalmasso, 1969 [82], *X*. *santos* Lamberti, Lemos, Agostinelli & D’Addabbo, 1993 [67], *X*. *sphaerocephalum*, *X*. *turcicum*, *X*. *turdetanense*, and *X*. *vallense* have been previously recorded within studies of dagger and needle nematodes infesting olives and vineyards in southern Spain [17, 18, 28]. Consequently, only D2-D3 sequences had been reported here for these samples. For other known species studied, representing the first molecular characterization and new records for olive or for Spain (*viz*. *X*. *cadavalense*, *X*. *conurum* and *X*. *pseudocoxi* Sturhan, 1985 [87]), a brief description and a morphometric comparison with previous records and paratypes is provided below (Figs 12, 13 and 14 and Table 9).

**Table 9:** Morphometrics of females of *Xiphinema cadavalense* Bravo & Roca, 1995, *Xiphinema conurum* Siddiqi, 1964 and *Xiphinema pseudocoxi* Sturhan, 1984 from the rhizosphere of cultivated and wild olives at several localities (Almería and Córdoba provinces) southern Spain^a^.

Host/locality, sample code	*Xiphinema cadavalense* cultivated olive (Espiel, Córdoba) ST077	*Xiphinema conurum* cultivated olive (Uleila del Campo, Almería) ST045	*Xiphinema pseudocoxi* wild olive (Alcaracejos, Córdoba) AR095
Characters/ratios^b^	Females	Females	Females
**n**	6	2	10
**L (mm)**	5.5 ± 0.25	4.0 ± 0.30	4.1 ± 0.29
	(5.2–5.9)	(3.8–4.2)	(3.8–4.8)
**a**	65.2 ± 5.7	117.1 ± 8.7	80.6 ± 7.8
	(55.0–70.9)	(111.0–123.3)	(70.3–91.9)
**b**	8.8 ± 0.7	11.7 ± 0.6	9.5 ± 0.8
	(8.0–10.1)	(11.3–12.0)	(8.3–10.9)
**c**	97.9 ± 13.8	74.9 ± 7.1	85.5 ± 12.3
	(77.8–112.5)	(69.9–79.9)	(70.2–104.9)
**c´**	1.1 ± 0.1	2.4 ± 0.0	1.4 ± 0.2
	(0.9–1.2)	(2.4–2.5)	(1.2–1.7)
**V**	50.3 ± 1.5	46.8 ± 1.1	42.9 ± 1.2
	(48.5–52.5)	(46.0–47.5)	(41.0–45.0)
**Odontostyle**	163.9 ± 2.6	102.5 ± 2.1	120.1 ± 4.2
	(161.0–167.0)	(101.0–104.0)	(114.5–126.0)
**Odontophore**	105.3 ± 5.4	63.0 ± 0.7	69.4 ± 2.2
	(98.5–111.5)	(62.5–63.5)	(67.0–74.5)
**Total stylet**	269.2 ± 8.0	165.5 ± 2.8	189.5 ± 4.8
	(259.5–278.5)	(163.5–167.5)	(181.5–197.0)
**Lip region diam.**	17.8 ± 0.9	13.0 ± 0.7	12.2 ± 0.4
	(17.0–19.5)	(12.5–13.5)	(11.5–12.5)
**Oral aperture-guiding ring**	156.8 ± 6.7	87.8 ± 0.4	106.6 ± 5.2
	(149.5–167.0)	(87.5–88.0)	(98.0–113.5)
**Tail length**	56.9 ± 8.0	53.3 ± 1.1	48.5 ± 5.7
	(48.0–67.5)	(52.5–54.0)	(39.5–56.0)
**J**	22.5 ± 4.1	12.5 ± 1.4	17.5 ± 2.7
	(15.5–28.0)	(11.5–13.5)	(14.5–22.0)

^a^ Measurements are in μm (except for L) and in the form: mean ± standard deviation (range).

^b^ Abbreviations as defined in Jairajpuri & Ahmad [51]. a, body length/maximum body width; b, body length/pharyngeal length; c, body length/tail length; c', tail length/body width at anus; V (distance from anterior end to vulva/body length) x 100; J (hyaline tail region length).

#### *Xiphinema cadavalense* Bravo & Roca, 1995

The amphimictic population of *Xiphinema* collected from the rhizosphere of cultivated olive (*Olea europaea* subsp. *europaea* L.) at Espiel (Córdoba province) corresponds fairly well with studied paratypes and original description of *X*. *cadavalense*. This population was characterised by a long body; lip region hemispherical, rounded both anteriorly and laterally and set off from body contour by slightly depression; long odontostyle and odontophore; reproductive system didelphic-amphidelphic with both branches about equally developed with a well-developed Z-differentiation with weakly muscularised wall and comprising 9–15 sclerotized bodies of variable size and petal shape, each one consisting of a large portion, irregularly spherical surrounded by a variable number of refractive pieces; spiniform structures and crystalloid bodies in very small size and low number present along the narrower and muscular tube-like of uterus; tail dorsally convex-conoid (dorsally convex and ventrally almost convex or slightly straight) ending in a terminal peg with blind canal (Fig 12 and Table 9). The observations on the general morphology nematode indicate that this *Xiphinema* population belongs to the *X*. non-*americanum* Group 5 in Loof and Luc [27], which agrees with the original description of *X*. *cadavalense* [52]. In addition, female morphometrics fit with those provided in the original description, except in having slightly longer body and odontostyle length (5.2–5.9 mm, 161.0–167.0 μm *vs* 4.0–5.3 mm, 150.5–164.5 μm, respectively), posterior guiding ring position from oral aperture (149.5–167.0 *vs* 126.5–148.5 μm) [52]. These differences may be due to geographical intraspecific variability. Up to our knowledge, this is the first report for Spain and confirms a wider distribution in the Iberian Peninsula, apart from original description in Portugal. According to the polytomous key of Loof & Luc [27], this Spanish population of *X*. *cadavalense* has the following specific alphanumeric codes (codes in parentheses are exceptions): A4-B2+3-C5a-D65-E56-F5-G4-H2-I3-J-K-L1.

D2-D3 segments of *X*. *cadavalense* (KX244900) was 98% similar (14 nucleotides and no indels) to *X*. *baetica* (KC567168), 97% similar (24 nucleotides and 3 indels) to *X*. *andalusiense* sp. nov. (KX244884-KX244888) and 96% similar (30 nucleotides and 10 indels) to *X*. *macrodora* (KU171040, KU171242). ITS1 sequence (KX244932) region also agrees with results obtained from D2-D3, this sequence was 90% similar (105 nucleotides and 28 indels) to *X*. *baetica* (KC567157), 89% (121 nucleotides and 35 indels) to *X*. *andalusiense* sp. nov. (KX244921-KX244925) and 86% (157 nucleotides and 70 indels) to *X*. *macrodora* (KU171048). The partial 18S region of *X*. *cadavalense* (KX244946), was very similar to several sequences of *Xiphinema* spp., including *X*. *diversicaudatum* (Micoletzky, 1927) Thorne, 1939 [88, 89] (JQ780346-JQ780349), *X*. *baetica* (KC567149) and *X*. *bakeri* Williams, 1961 [90] (AY283173).

#### *Xiphinema conurum* Siddiqi, 1964

The Spanish population of this species from the rhizosphere of olive was characterised by a lip region rounded offset from the rest of the body by a conspicuous depression, two equally developed female genital branches, vulva slightly anterior to mid-body, uterus with uterine differentiation, presence of Z-differentiation with small granular bodies plus small spines (in low number), female tail conical, ventral profile nearly straight, dorsal profile regularly curved with rounded terminus (Fig 13). The morphology and morphometric of this population agree closely with the original description and redescription of the species by Siddiqi [53] and Luc & Aubert [91], likewise recently examined specimens from Soukra, Tunisia by Guesmi-Mzoughi *et al*. [92]. Up to our knowledge, this is the first report of this species for Spain.

D2-D3 sequence for *X*. *conurum* (KX244902) matched well, 99% similar with former sequences from Tunisia deposited in GenBank (KX062671-KX062673); and ITS1 (KX244934) was 95–96% similar with former sequences from Tunisia deposited in GenBank (KX062696-KX062697). And partial 18S (KX244947) was provided for the first time in this research, being 99% similar to several *Xiphinema* spp. such as *X*. *nuragicum* (GU725081) or *X*. *israeliae* Luc, Brown & Cohn, 1982 [93] (KJ802900), extending the molecular diversity of this species to newly studied area.

#### *Xiphinema pseudocoxi* Sturhan, 1984

The amphimictic population of *Xiphinema* collected from the rhizosphere of wild olive (*Olea europaea* subsp. *silvestris* (Miller) Lehr) at Alcaracejos (Córdoba province) agrees fairly well with original description of *X*. *pseudocoxi*. This population was characterised by a moderately long body in an open C-shaped after fixation; lip region distinct from the body contour by a depression, frontally rounded; female reproductive system didelphic-amphidelphic having both branches about equally developed; Z-differentiation with weakly muscularised wall formed by 6–10 globular bodies similar in size, and irregularly spherical surrounded by a variable number of refractive pieces; no spiniform structures and, crystalloid bodies nor sperm cells observed along uterus; female tail convex-conoid, varying slightly in shape, and ending in a terminal peg with a blind canal (Fig 14, Table 9). Based on the morphological character observations we confirm that this *Xiphinema* population belongs to the *X*. non-*americanum* Group 5 in Loof and Luc [27], which is in agreement with the original description of *X*. *pseudocoxi* [87]. Additionally, female morphometrics fit with those provided in the original description and rather similar to data reported subsequently for other populations of Spain and Portugal, except for minor differences in nematode body and odontostyle length, which may be due to few specimens originally studied or geographical intraspecific variability [87, 94, 95]. This new Spanish population extends the species distribution in Europe, and confirms a wider distribution in the Iberian Peninsula, apart from other populations from Spain, Portugal, and original description in Germany. According to the polytomous key of Loof & Luc [27], the new Spanish population of *X*. *pseudocoxi* has the following specific alphanumeric codes (codes in parentheses are exceptions): A4-B2-C5a-D45-E4(5)-F4(5)-G2-H2-I3-J-K-L1.

Sequences for *X*. *pseudocoxi* (KX244915-KX244916) were obtained for the first time in this study. The closet species regarding D2-D3 segments of *X*. *pseudocoxi* (KX244915-KX244916) were *X*. *globosum* Sturhan, 1978 [96] (GU549474, 97% similar, 20 nucleotides and 3 indels), *X*. *diversicaudatum* (JQ780360-JQ780366, 96% similar) and *X*. *coxi europaeum* (KC567174-KC567176, 96% similar). Similarly, ITS1 region (KX244939-KX244940) also showed some similarity with *X*. *globosum* (GU549475, 88% similar, 127 nucleotides and 35 indels), *X*. *diversicaudatum* (HG969304, 87% similar, 154 nucleotides and 46 indels) and *X*. *coxi europaeum* (KC567160, 86% similar, 154 nucleotides and 43 indels). Finally, the partial 18S of *X*. *pseudocoxi* (KX244948) matched closely (99%) with several *Xiphinema* spp., such as *X*. *globosum* (GU549476), *X*. *diversicaudatum* (EF538761), *X*. *bakeri* (AY283173), *X*. *vuittenezi* Luc, Lima, Weischer & Flegg, 1964 [97] (EF614267) and *X*. *index* (AY687997).

#### Phylogenetic relationships of the *Xiphinema* spp

The amplification of D2-D3 expansion segments of 28S rRNA, ITS1 rRNA, and partial 18S rRNA yielded a single fragment of approximately 800 bp, 1000 bp, and 1800 bp, respectively, based on gel electrophoresis. Sequences from other species of *Xiphinema* spp. obtained from National Center for Biotechnology Information (http://www.ncbi.nlm.nih.gov/) were used for further phylogenetic studies. Sequences for *X*. *andalusiense* sp. nov., *X*. *cadavalense*, *X*. *celtiense* sp. nov., *X*. *duriense*, *X*. *iznajarense* sp. nov., *X*. *mengibarense* sp. nov., *X*. *opisthohysterum* and *X*. *pseudocoxi* were obtained for these species in this study. On the other hand, sequences from *X*. *adenohystherum*, *X*. *cohni*, *X*. *conurum*, *X*. *hispanum*, *X*. *hispidum*, *X*. *incertum*, *X*. *index*, *X*. *italiae*, *X*. *nuragicum*, *X*. *parapachydermum*, *X*. *turcicum* and *X*. *turdetanense* matched well with former sequences deposited in GenBank, and spread out the molecular diversity of these species to the newly studied areas.

**Fig 15:**
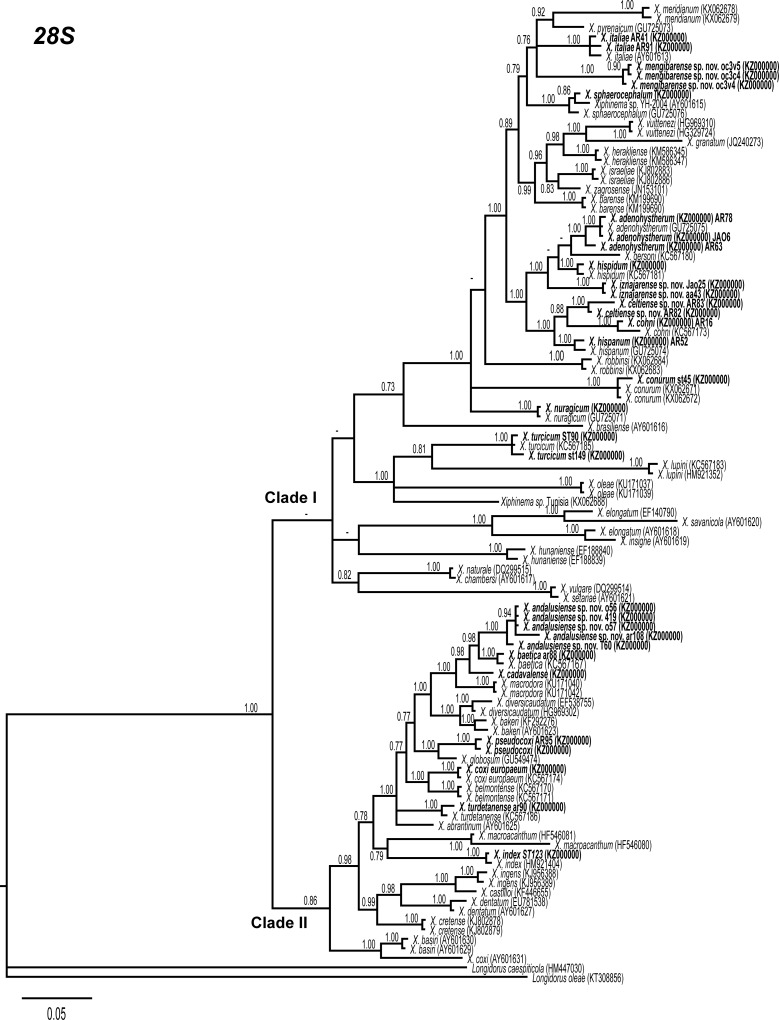
The 50% majority rule consensus tree from Bayesian inference analysis generated from the D2-D3 of 28S rRNA gene dataset of *Xiphinema* spp. with the GTR+I+G model. Posterior probabilities more than 0.70 are given for appropriate clades. Newly obtained sequences are in bold letters. Scale bar = expected changes per site.

**Fig 16:**
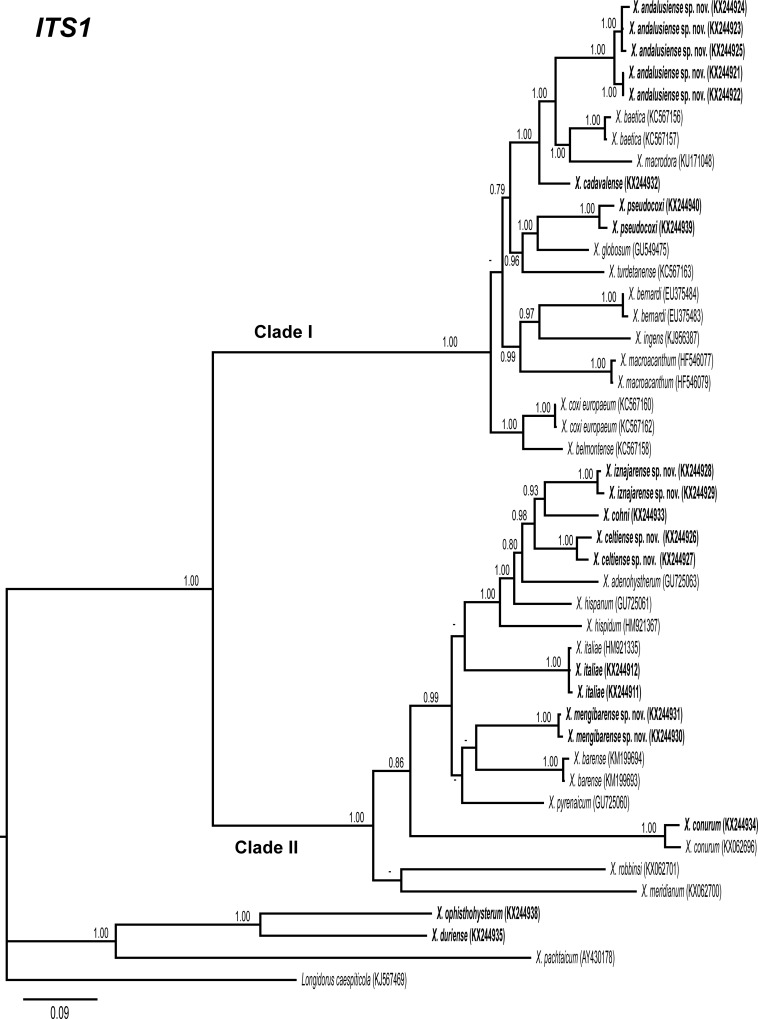
The 50% majority rule consensus trees from Bayesian inference analysis generated from the ITS rRNA gene dataset of *Xiphinema* spp. with the GTR+I+G model. Posterior probabilities more than 0.70 are given for appropriate clades. Newly obtained sequences are in bold letters. Scale bar = expected changes per site.

**Fig 17:**
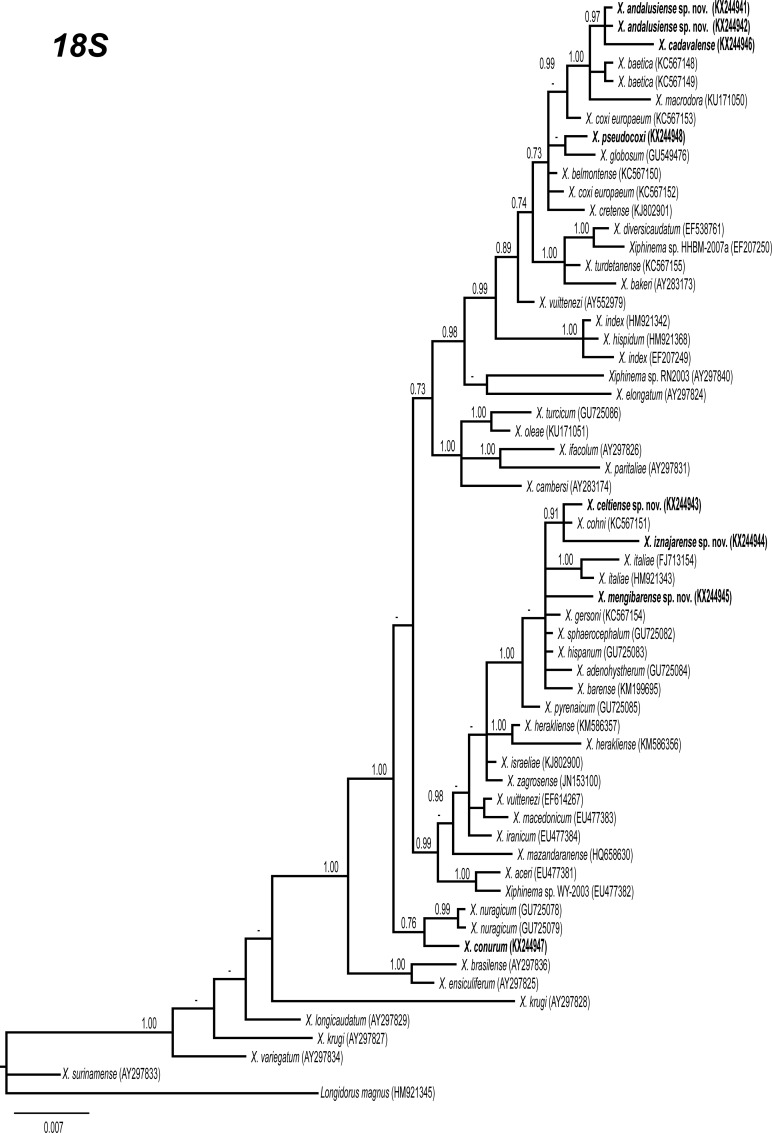
The 50% majority rule consensus trees from Bayesian inference analysis generated from the partial 18S rRNA gene dataset of *Xiphinema* spp. with the TIM3+I+G model. Posterior probabilities more than 0.70 are given for appropriate clades. Newly obtained sequences are in bold letters. Scale bar = expected changes per site.

Phylogenetic relationships among *Xiphinema* non-*americanum*-group species inferred from analyses of D2-D3 expansion segments of 28S, ITS1, and the partial 18S rDNA gene sequences using BI are given in Figs 15, 16 and 17, respectively. Poorly supported clusters were not explicitly labelled. The 50% majority rule consensus 28S rRNA gene BI tree of *X*. non-*americanum*-group spp. based in a multiple edited alignment including 103 sequences and 753 total characters showed two clearly separated (PP = 1.00) major clades (Fig 15). Clade I was not well supported. This clade grouped thirty-five species including morphospecies from Groups 1, 4, 5, 6, 7 and 8. This major clade grouped three of the four new species described in this study: *X*. *celtiense* sp. nov. from wild olive, and *X*. *iznajarense* sp. nov. and *X*. *mengibarense* sp. nov. from cultivated olive. *Xiphinema celtiense* sp. nov. formed a well-supported subclade (PP = 1.00) with *X*. *cohni* (KC567173, (KX244901) and *X*. *hispanum* (GU725074, KX244905), this clade was related (PP = 1.00) with another subclade which was formed by *X*. *iznajarense* sp. nov. (KX244891-KX244892), *X*. *adenohystherum* (KX244896-KX244898, GU725075), *X*. *hispidum* (KC567181, KX244906) and *X*. *gersoni* (KC567180). Finally, *X*. *mengibarense* sp. nov. formed a low-supported subclade (PP = 0.76) with *X*. *italiae* (AY601613, KX244911-KX244912), *X*. *pyrenaicum* (GU725073), and *X*. *meridianum* Heyns, 1971 [98] (KX062678-KX062679). Clade II was moderately supported (PP = 0.86) and was formed by twenty species, all of them from the morphospecies Group 5, except *X*. *bakeri* and *X*. *index* which belong to Groups 7 and 8, respectively. This clade grouped sequences from the new species *X*. *andalusiense* sp. nov. (KX244884-KX244888) and the new accessions from *X*. *cadavalense* (KX244900), *X*. *conurum* (KX244902), and *X*. *pseudocoxi* (KX244915-KX244916). *Xiphinema andalusiense* sp. nov. (KX244884-KX244888) from wild olive occupied a superior position within the clade II forming a well-supported subclade (PP = 1.00) with *X*. *cadavalense* (KX244900) from cultivated olive, *X*. *baetica* (KC567167, KX244899) and *X*. *macrodora* (KU171040, KU171042). Finally, *X*. *pseudocoxi* (KX244915-KX244916) was phylogenetically related to *X*. *globosum* (GU549474) forming a well-supported clade (PP = 0.99).

Difficulties were experienced with alignment of the ITS1 sequences due to scarce similarity, thus, only related sequences were used. The alignment generated for the 45 sequences of ITS1, comprising several *X*. non-*americanum*-group species, was 1113 characters after discarding ambiguously aligned regions from the alignment. Two new accessions were used as outgroup, *X*. *duriense* (KX244935) and *X*. *opisthohysterum* (KX244938). The 50% majority rule consensus BI tree of *X*. non-*americanum-*group spp. showed two major clades (PP = 1.00) similar to those obtained for D2-D3 region (Fig 16). Clade I was formed by twelve *Xiphinema* species from morphospecies Group 5 including *X*. *andalusiense* sp. nov. (KX244921-KX244925), *X*. *pseudocoxi* (KX244939-KX244940) and *X*. *cadavalense* (KX244932). *Xiphinema andalusiense* sp. nov. (KX244921-KX244925) and *X*. *cadavalense* (KX244932) clustered with *X*. *baetica* (KC567156-KC567157) and *X*. *macrodora* (KU171048) in a well-supported subclade (PP = 1.00), these results agree with the results obtained with D2-D3 region. *Xiphinema pseudocoxi* and *X*. *globosum* were also phylogenetically related to this marker and they were placed in a well-supported subclade (PP = 1.00) which was related (PP = 0.96) at the same time with *X*. *turdetanense* (KC567163). Clade II grouped thirteen species from different morphospecies Groups 1, 4, 5 and 7, including *X*. *celtiense* sp. nov., *X*. *iznajarense* sp. nov. and *X*. *mengibarense* sp. nov. *Xiphinema iznajarense* sp. nov. (KX244928-KX244929), and *X*. *celtiense* sp. nov. (KX244926-KX244927) clustered together with *X*. *cohni* (KX244933), *X*. *adenohystherum* (GU725063), *X*. *hispanum* (GU725061) and *X*. *hispidum* (HM921367) as occurred in the D2-D3 tree. Finally, *X*. *mengibarense* sp. nov. (KX244930-KX244931) formed a low-supported subclade with *X*. *barense* Lamberti, Roca, Agostinelli & Bleve-Zacheo, 1986 [99] (KM199694-KM199693) and this subclade was related to *X*. *pyrenaicum* (GU725060) although this relation also was poorly supported. The new accessions for *X*. *duriense* (KX244935) and *X*. *opisthohysterum* (KX244938) clustered together with *X*. *pachtaicum* (AY430178) as an outgroup, all of them from the *X*. *americanum*-group (Fig 17).

The 50% majority rule BI tree of a multiple alignment including 60 18S sequences and 1647 bp long showed several major clades (Fig 17). Additionally, in the D2-D3 and ITS1 trees, *X*. *andalusiense* sp. nov. (KX244941-KX244942) clustered with *X*. *cadavalense*, *X*. *macrodora* and *X*. *baetica* within the same well-supported subclade (PP = 1.00). Phylogenetic inferences based on 18S also suggest that *X*. *pseudocoxi* and *X*. *globosum* are related species, although this relation was poorly supported (Fig 17). Finally, *X*. *iznajarense* sp. nov. (KX244944), *X*. *celtiense* sp. nov. (KX244943) and *X*. *mengibarense* sp. nov. (KX244945) clustered in this case with *X*. *cohni* (KC567151), *X*. *hispanum* (GU725083), *X*. *adenohystherum* (GU725084), *X*. *italiae* (FJ713154, HM921343), *X*. *barense* (KM199695), *X*. *gersoni* (KC567154), X. *sphaerocephalum* (GU725082), and *X*. *pyrenaicum* (GU725085) within a well-supported subclade (PP = 1.00).

## Discussion

This study aimed to get knowledge and a better understanding on the occurrence, abundance and biodiversity of dagger nematodes of the genus *Xiphinema* associated with wild and cultivated olives in southern Spain, as well as their distribution and molecular phylogeny. This was conducted in an extensive and systematic nematological survey that included 211 locations and 453 sampling sites. We found 385 Spanish populations of *Xiphinema* spp. infesting olive soils. We described four new *Xiphinema* species, enlarging the diversity of *Xiphinema* species in the Iberian Peninsula which is in agreement with previous data obtained for the phylogeny and biogeography of the genus *Xiphinema* and *Longidorus* in the Euro-Mediterranean region [13, 17, 18, 28, 31, 100, 101]. To the date, to our knowledge, this work is the largest phylogenetic analysis of the genus *Xiphinema* based on nuclear rDNA markers.

The genus *Xiphinema* is one of the most diverse PPN associated with olive, with twenty species (*viz*. *X*. *aequum* Roca & Lamberti, 1988 [102], *X*. *barense*, *X*. *californicum* Lamberti & Bleve-Zacheo, 1979 [103], *X*. *cretense* Tzortzakakis et al., 2014 [33], *X*. *diversicaudatum*, *X*. *duriense*, *X*. *elongatum* [104], *X*. *herakliense*, *X*. *incertum*, *X*. *index*, *X*. *ingens* Luc, 1963 [72], *X*. *italiae*, *X*. *israeliae*, *X*. *lusitanicum* Sturhan, 1983 [105], *X*. *macroacanthum* Lamberti, Roca & Agostinelli, 1990 [106], *X*. *macrodora*, *X*. *madeirense*, *X*. *nuragicum*, *X*. *oleae*, *X*. *opisthohysterum*, *X*. *pachtaicum*, *X*. *parapachydermum*, *X*. *plesiopachtaicum*, *X*. *rivesi*, *X*. *sahelense* Dalmasso, 1969 [82], *X*. *turcicum*, *X*. *vallense*, *X*. *vuittenezi* and several unidentified species) reported in various countries of the Mediterranean Basin [18, 28, 33, 34, 42, 80]. The present results increase the previous data about diversity of *Xiphinema* species detected in olive worldwide, including four new species from the *X*. non-*americanum*-group. All these species were new records for olive with the exception of *X*. *pachtaicum*, *X*. *index*, *X*. *italiae*, *X*. *nuragicum* and *X*. *turcicum* [34]. In addition to the remarkable prevalence of *Xiphinema* spp. observed in both olive types, our study showed a great species diversity, that was mainly associated with the *X*. non-*americanum*-group species (*P* < 0.05, Fig 2D), being widely distributed in Andalusia but in particular mainly associated with wild olive in Cádiz province, a more humid and ecologically diverse area than the rest of the Andalusian provinces. However, *X*. *pachtaicum* was present in the majority of the sampled localities in wild and cultivated olives showing the plasticity of this species for a wide diversity of ecological conditions (Fig 1). Overall, *X*. *pachtaicum* was detected in 74.2% of the total sampling sites, specifically 67 out of 115 and 268 out of 338 associated with wild and cultivated olive, respectively. As reported in previous studies, this species is widespread in the Mediterranean Basin [16, 23, 28, 31, 33, 92, 107, 108], including olive [9, 109, 110]. In Spain, *X*. *pachtaicum* was also the most prevalent dagger nematode in vineyards and stone-fruit orchards [31, 111]. The widespread distribution of *X*. *pachtaicum* may suggest also adaptability to a range of soil types, and reproduction sustained over a broad range of temperatures [112, 113]. Nevertheless, these wider ecological requirements are difficult to explain regarding their low genetic diversity [108] and could be more associated with the presence of specific ovarial-intestine endosymbionts [114], but some of the other species from the *X*. *americanum*-group also possesses ovarial-intestine endosymbionts and were more restricted to some areas (*viz*. *X*. *opisthohysterum*, *X*. *santos*, *X*. *incertum*, *X*. *madeirense*, *X*. *vallense*, *X*. *plesiopachtaicum* and *X*. *rivesi*) [114]. Other species with a broad distribution were included in the *X*. non-*americanum*-group, *i*.*e*. *X*. *italiae* found in all provinces, *X*. *nuragicum* in 7 out of 8 provinces, and *X*. *coxi europaeum* in 5 out of 8 provinces. In this sense, the presence of a high number of frequent species belonging to *X*. non-*americanum*-group (*i*.*e*. *X*. *italiae*, *X*. *nuragicum*, *X*. *coxi europaeum* or to a lesser extent *X*. *adenohystherum*) explains the higher value observed in Hill´s 2 (Dominance diversity) index with respect to *X*. *americanum*-group (*P* < 0.01, Fig 2D).

Nematodes of the genus *Xiphinema* cause damage to olive by feeding on unmodified plant root cells and causing cell necrosis and galling in root apex [54, 115]. However, some species are also capable to transmit pathogenic viruses to olive, specifically species belonging to the Nepovirus genus [24], such as *X*. *diversicaudatum* and *X*. *vuittenezi* [116]. Nevertheless, some dagger nematodes have been considered as major pathogens on olive trees in several countries including Chile or USA, where it was reported that *Xiphinema* spp. were responsible for 5 to 10% of loss production resulting in an estimated $39 million loss [117, 118]. Although our results mainly revealed low densities of *Xiphinema* spp. in both olive types studied (Table 2 and S1 Table), in some sampling sites the densities were high, *i*.*e*. 414 or 350 nematodes per 500 cm^3^ of soil for *X*. *pachtaicum* and *X*. *italiae*, respectively. In this regard, similar nematode densities of *Xiphinema* spp. have been reported to reduce plant-growth by feeding directly on olive roots, *e*.*g*. 65% in the case of *X*. *elongatum* [119], and in several plants including other crops [120, 121] or ornamental plants [122]. On the other hand, total abundance of nematodes in each sampling site resulted significantly higher in *X*. *americanum*-group in comparison to *X*. non-*americanum*-group (*P <* 0.001, Fig 2D). We found a significant increase in the abundance in cultivated than in wild olive (*P <* 0.01, Fig 2B) for the *X*. *americanum*-group, mainly because of the prevalence and high average nematode density detected for *X*. *pachtaicum* on cultivated olive (Table 2 and S1 Table). Overall, these results could support the hypothesis that *X*. *pachtaicum* could be a real problem in olive orchards [123], although more studies would be required to clarify it. In general, *Xiphinema* spp. are difficult to culture under glasshouse conditions, and it is possible, that these nematodes are more pathogenic to olive in the field than is indicated by glasshouse test, since their population densities in such situations are likely to exceed those that can be attained in glasshouses [117].

Overall, nematode diversity decreases rapidly to agricultural management including plant-parasitic nematodes [124]. Our results showed lower diversity indexes values, specifically for Richness diversity, in wild than in cultivated olives (*P <* 0.001, Fig 2A). These differences were emphasized when *X*. *americanum*-group and *X*. non-*americanum*-group species were analyzed separately (*P <* 0.05, Fig 2B and 2C). This fact showed the effect of agricultural management to a wide range of changes in physical, chemical and biological properties of the soil, and alterations in the autoregulation in nematode assemblages, when compared natural (wild olive) with agricultural ecosystems (cultivated olive). In this sense, several papers showed the effect of these parameters or agricultural practices in the olive nematode community [110, 125, 126]. However, according to the higher number of species identified from *X*. non-*americanum*-group likewise the high prevalence of this group of nematodes associated to wild olive resulted in a higher value of Richness diversity in this type of olive in comparison to cultivated olive (*P <* 0.05, Table 2 and Fig 2C) in contrast to observed in *X*. *americanum*-group showing the possible plasticity of this species for a wide diversity of ecological requirements as discussed above. On the other hand, the distribution of the 385 *Xiphinema* populations collected in Andalusia did not revealed geographic associations to certain areas (Fig 1). Although agricultural activities may result in the widespread dissemination of *Xiphinema* species [112], the geographical distribution of *Xiphinema* species in wild and cultivated olives in southern Spain suggest a pattern linked to ecological factors. As previously reported by Archidona-Yuste *et al*. [13] for *Longidorus* species: “longidorids could have a lower dissemination level by human activities than other plant-parasitic nematodes (i.e. cyst- or root-lesion nematodes) because of their sensitivity to fast desiccation, large body size, and the absence of survival-resistance forms”. Unfortunately, little is known about the ecological requirements of *Xiphinema* nematodes and further research is needed [112]. Some provinces as Cádiz, Córdoba and Jaén have showed a higher diversity than other with 17, 15 and 12 species, respectively. Some of these provinces as Cádiz showed more favorable environment for nematodes development due to the higher relative humidity and water content in the soil. By contrast, *Longidorus* spp. showed evidence of some geographic species associations in Andalusia [13]. Consequently, further research is needed in order to determine the influence of physico-chemical soil factors on the prevalence and distribution of *Xiphinema* spp. in southern Spain and other wider areas.

Sequences of nuclear ribosomal RNA genes, particularly D2-D3 and ITS1, are useful molecular markers for providing accurate species identification of Longidoridae [13, 16, 18, 28, 30, 127]. The majority of the identified species in the rhizosphere of olive matched former molecularly characterized species in other studies. In this sense, this study provides new molecular markers for partial 18S (*X*. *cadavalense*, *X*. *pseudocoxi*, and *X*. *conurum*) and for ITS1 (*X*. *cadavalense*, *X*. *pseudocoxi*, *X*. *cohni*, *X*. *opisthohysterum* and *X*. *duriense*). D2-D3 expansion region was more useful for establishing phylogenetic relationships among *Xiphinema* species than ITS1 or 18S. Phylogenetic analyses based on D2-D3, ITS1, and partial 18S using BI resulted in a consistent position for the newly described species of *X*. non-*americanum-*group species from Spain, which grouped in two separated clades, and mostly agree with the clustering obtained by other authors [17, 18]. These species showed a good congruence between morphometric characters and phylogenetic positions as it is the case of *X*. *andalusiense* sp. nov., *X*. *baetica*, and *X*. *cadavalense*. In the case of *X*. *andalusiense* sp. nov. *vs X*. *baetica*, only lower a and c’ ratios, the absence of spines in the uterus, the absence of males and different ribosomal genes could separate *X*. *baetica* from *X*. *andalusiense* sp. nov. These species probably evolved in the Iberian Peninsula as they occur only there. The Iberian Peninsula has been suggested as a possible center of recent speciation for PPN nematode genera such as *Longidorus*, *Trichodorus* or *Rotylenchus* species [33]. *Xiphinema celtiense* sp. nov., *X*. *iznajarense* sp. nov. and *X*. *mengibarense* sp. nov. could be clearly separated morphologically and molecularly from the other *Xiphinema* species. The majority of the species showed congruence in the phylogenetic relationships within D2-D3, ITS1, and partial 18S using the DNA from the same individual and these markers matched very well with the sequences deposited in the GenBank. This result is in contrast with the close related genus *Longidorus* found in a similar sampling scheme and localities in which the diversity of species was lower and all the species occupies two major positions in the phylogenetic clade [28].

## Conclusions

In summary, this study provides new insights into the diversity of this genus associated with the olive in Mediterranean conditions with important differences related to the species within the *X*. *americanum-*group and the *non-americanum* group species. This research provides molecular markers for precise and unequivocal diagnosis of some species of *Xiphinema* in order to differentiate virus vector or quarantine species. Furthermore, it reflects that similar intensive and extensive integrative studies on *Xiphinema* species based on widest areas may help to elucidate the evolutionary origin of *Xiphinema* species. In this sense, further studies based on widespread species (i.e. *X*. *pachtaicum*) could also help to clarify if the main speciation occurred in Africa leading to many apomictic species in tropical and subtropical environments as hypothesised by Coomans [128], or in South America but in this case information is limited.

## Supporting Information

S1 Table Average soil nematode population density (number of specimens) and prevalence (%) of *Xiphinema* spp. in wild and cultivated olives in provinces of Andalusia, southern Spain. (DOCX)
