# Supplementary material for: Remarkable Diversity and Prevalence of Dagger Nematodes of the Genus Xiphinema Cobb, 1913 (Nematoda: Longidoridae) in Olives Revealed by Integrative Approaches
Source: PLoS One. 2016 Nov 9;11(11):e0165412. doi: 10.1371/journal.pone.0165412 (PMC5102458; doi:10.1371/journal.pone.0165412)
Supplement: S1 Table — (DOCX) [file pone.0165412.s001.docx]

**Table S1. Average soil nematode population density (number of specimens) and prevalence (%) of *Xiphinema* spp. in wild and cultivated olives in provinces of Andalusia, southern Spain.^a^**

|  | **Andalusian provinces** | | | | | | | | | | | | | | | |
| --- | --- | --- | --- | --- | --- | --- | --- | --- | --- | --- | --- | --- | --- | --- | --- | --- |
|  | **Almería** | | **Cádiz** | | **Córdoba** | | **Granada** | | **Huelva** | | **Jaén** | | **Málaga** | | **Seville** | |
| **Host plant^b^** | **W** | **C** | **W** | **C** | **W** | **C** | **W** | **C** | **W** | **C** | **W** | **C** | **W** | **C** | **W** | **C** |
| **Number of samples** | 8 | 25 | 53 | 19 | 23 | 79 | 1 | 39 | 9 | 20 | 6 | 63 | 8 | 28 | 7 | 65 |
| ***Xiphinema* spp.** | **12.6 (50.0)** | **36.6 (52.0)** | **23.3 (100)** | **39.3 (100)** | **25.5 (95.7)** | **38.0**  **(83.4)** | **11.0 (100)** | **33.2 (84.6)** | **19.4**  **(100)** | **47.2 (95.0)** | **28.6**  **(100)** | **32.5 (79.4)** | **20.5 (87.5)** | **42.0 (85.7)** | **15.2 (85.7)** | **39.2 (81.5)** |
|  |  |  |  |  |  |  |  |  |  |  |  |  |  |  |  |  |
| ***X. americanum-*group spp.^c^** | **15.0 (50.0)** | **45.4 (52.0)** | **19.4 (83.0)** | **49.9 (100)** | **27.3 (78.3)** | **44.3 (81.0)** | **11.0**  **(100)** | **31 (79.5)** | **18.6 (55.6)** | **48.3 (90.0)** | **45.6 (83.3)** | **35.4 (77.8)** | **24.9 (87.5)** | **43.6 (82.1)** | **22.2 (85.7)** | **47.0**  **(81.5)** |
| *Xiphinema duriense* | - | - | - | - | - | - | - | - | 2.0 (11.1) | 1.0  (5.0) | - | - | - | - | - | - |
| *Xiphinema incertum* | - | - | 17.9 (13.2) | - | 38.5 (8.7) | - | - | - | 28.0 (11.1) | - | - | - | 22.0  (12.5) | - | - | 38.0 (1.5) |
| *Xiphinema madeirense* | - | - | 11.0 (1.9) | - | - | - | - | - | - | - | - | - | - | - | - | - |
| *Xiphinema opisthohysterum* | - | - | 3.0 (1.9) | - | - | - | - | - | - | - | 14.0 (16.1) | - | - | - | - | - |
| *Xiphinema pachtaicum* | 15.0 (50.0) | 45.4 (52.0) | 20.7 (52.8) | 54.1 (100) | 25.9 (69.6) | 44.8 (81.0) | 11.0  (100) | 38.7 (79.5) | 21.0 (33.3) | 53.2 (85.0) | 43.0 (33.3) | 35.4 (77.8) | 25.3 (87.5) | 43.6 (82.1) | 2.2 (85.7) | 47.2 (81.5) |
| *Xiphinema parapachydermum* | - | - | 31.8 (7.5) | - | - | 8.0 (1.3) | - | - | - | - | 16.0 (16.1) | - | - | - | - | - |
| *Xiphinema plesiopachtaicum* | - | - | - | - | - | - | - | - | - | - | 112 (16.1) | - | - | - | - | - |
| *Xiphinema santos* | - | - | 9.0  (1.9) | - | - | - | - | - | - | - | - | - | - | - | - | - |
| *Xiphinema rivesi* | - | - | - | - | - | - | - | - | - | 58.0  (5.0) | - | - | - | - | - | - |
| *Xiphinema vallense* | - | - | 13.6 (13.2) | 14.0 (10.5) | - | - | - | - | - | - | - | - | - | - | - | - |
|  |  |  |  |  |  |  |  |  |  |  |  |  |  |  |  |  |
| ***X. non-americanum-group spp.*^c^** | **3 (12.5)** | **13.8 (20.0)** | **26.7 (84.9)** | **16.9 (52.6)** | **23.9 (73.9)** | **16.4 (21.5)** | **-** | **4.7 (15.4)** | **19.8 (88.9)** | **42.8 (30.0)** | **11.6 (66.7)** | **8.3 (9.5)** | **8.7 (37.5)** | **38.8 (39.3)** | **4.8 (42.9)** | **23.0 (36.9)** |
| ***Xiphinema andalusiense* sp. nov.** | - | - | - | - | 12.5  (8.7) | - | - | - | - | - | 16.0 (16.1) | - | - | - | - | - |
| ***Xiphinema celtiense sp. nov.*** | - | - | - | - | 82.0  (4.4) | - | - | - | - | - | - | - | - | - | 3.0 (14.3) | - |
| ***Xiphinema iznajarense sp. nov.*** | - | - | - | - | - | 34.0  (1.3) | - | - | - | - | - | - | - | - | - | - |
| ***Xiphinema mengibarense* sp. nov.** | - | - | - | - | - | - | - | - | - | - | - | 12.0  (1.6) | - | - | - | - |
| *Xiphinema adenohystherum* | - | - | 3.3 (15.1) | - | 10.3 (17.4) | 1.0  (1.3) | - | - | - | - | 14.0 (16.7) | - | - | - | - | - |
| *Xiphinema baetica* | - | - | 1  (1.9) | - | - | - | - | - | - | - | - | - | - | - | - | - |
| *Xiphinema cadavalense* | - | - | - | - | - | 1.0 (1.3) | - | - | - | - | - | - | - | - | - | - |
| *Xiphinema cohni* | - | - | 32 (1.9) | - | - | - | - | - | - | - | - | - | - | - | - | - |
| *Xiphinema conurum* | - | 3 (7.70) | - | - | - | - | - | - | - | - | - | - | - | - | - | - |
| *Xiphinema coxi europaeum* | - | - | 10.0 (3.8) | - | 1.3 (13.0) | 1.0 (2.5) | - | - | 31.7 (33.3) | - | - | - | - | - | 10.0 (14.3) | - |
| *Xiphinema hispanum* | - | - | - | - | - | - | - | - | - | - | 6.5 (33.3) | - | - | - | - | - |
| *Xiphinema hispidum* | - | - | 5.3 (7.6) | - | - | - | - | - | 12.0 (11.1) | - | - | - | - | - | - | - |
| *Xiphinema index* | - | - | - | - | - | 3.0 (1.3) | - | - | - | - | - | - | - | - | - | - |
| *Xiphinema italiae* | 3 (12.5) | 16.5 (16.0) | 60.4 (17.0) | 13.6 (26.3) | - | 7.7 (3.8) | - | 3.0 (5.1) | 20.5 (22.2) | 51.5 (20.0) | - | 13.5 (3.2) | 9.0 (12.5) | 59.0 (3.6) | - | 19.5 (21.5) |
| *Xiphinema lupini* | - | - | - | - | - | - | - | - | 8.0 (22.2) | - | - | - | - | - | 4.0 (14.3) | - |
| *Xiphinema macrodora* | - | - | - | - | 7.0  (4.4) | 14.0 (1.3) | - | - | - | 8.0  (5.0) | - | - | - | - | - | - |
| *Xiphinema nuragicum* | - | - | 35.7 (49.1) | 23.0 (21.1) | 40.4 (30.4) | 25.9 (11.4) | - | 6.3 (2.6) | 14.0 (11.1) | - | - | 2.5 (3.2) | 8.5 (25.0) | 38.4 (32.1) | - | 29.4 (16.9) |
| *Xiphinema oleae* | - | - | 4  (1.9) | - | - | - | - | - | - | - | - | - | - | - | - | - |
| *Xiphinema pseudocoxi* | - | - | - | - | 10.0  (4.4) | - | - | - | - | - | - | - | - | - | - | - |
| *Xiphinema sphaerocephalum* | - | - | - | - | - | - | - | - | - | - | 15.0 (16.1) | - | - | - | - | - |
| *Xiphinema turcicum* | - | - | 2.3  (5.7) | 15.0 (5.3) | - | - | - | 3.0 (2.6) | - | - | - | 6.0 (1.6) | - | 22.0 (3.6) | 2.0 (14.3) | 1.0 (1.5) |
| *Xiphinema turdetanense* | - | - | 2.2 (9.4) | - | - | - | - | - | - | - | - | - | - | - | - | - |

^a^ Population density was calculated as the mean of *Xiphinema* nematodes per 500 cm^3^ of soil. The prevalence was computed by dividing the numbers of samples in which the *Xiphinema* species was observed by the total number of samples and expressed as a percentage

**^b^** Host plant: W = wild olive; C = cultivated olive.

^c^ *Xiphinema* group species established by Loof & Luc, 1990; Lamberti et al., 2000; and Coomans et al., 2001

(-) not found
